# Design, Synthesis and Biological Evaluation of Novel 4-Substituted Coumarin Derivatives as Antitumor Agents

**DOI:** 10.3390/molecules23092281

**Published:** 2018-09-06

**Authors:** Ran An, Zhuang Hou, Jian-Teng Li, Hao-Nan Yu, Yan-Hua Mou, Chun Guo

**Affiliations:** 1 School of Pharmaceutical Engineering, Shenyang Pharmaceutical University, Shenyang 110016, China; bear2015a@163.com (R.A.); houzhuang8@sina.com (Z.H.); jtli2014@outlook.com (J.-T.L.); yhna380@hotmail.com (H.-N.Y.); 2 Department of Pharmacology, Shenyang Pharmaceutical University, Shenyang 110016, China

**Keywords:** anticancer, coumarin, hypoxia, 1,2,3-triazole

## Abstract

Herein, fifteen new compounds containing coumarin, 1,2,3-triazole and benzoyl- substituted arylamine moieties were designed, synthesized and tested in vitro for their anticancer activity. The results showed that all tested compounds had moderate antiproliferative activity against MDA-MB-231, a human breast cancer cell line, under both normoxic and hypoxic conditions. Furthermore, the 4-substituted coumarin linked with benzoyl 3,4-dimethoxyaniline through 1,2,3-triazole (compound **5e**) displayed the most prominent antiproliferative activities with an IC_50_ value of 0.03 μM, about 5000 times stronger than 4-hydroxycoumarin (IC_50_ > 100 μM) and 20 times stronger than doxorubicin (IC_50_ = 0.60 μM). Meanwhile, almost all compounds revealed general enhancement of proliferation-inhibiting activity under hypoxia, contrasted with normoxia. A docking analysis showed that compound **5e** had potential to inhibit carbonic anhydrase IX (CA IX).

## 1. Introduction

Coumarin, a significant scaffold of both natural and synthetic origin, displays versatile pharmacological properties that include antibacterial [1], antioxidant [2], anticoagulant [3], anti-Alzheimer [4], anti-HIV [5], antimicrobial [6,7] and anticancer activities. Their antitumor effects are widely reported to be related to the inhibition of the cellular proliferation through binding to different targets and diverse pharmacological mechanisms.

**Figure 1:**
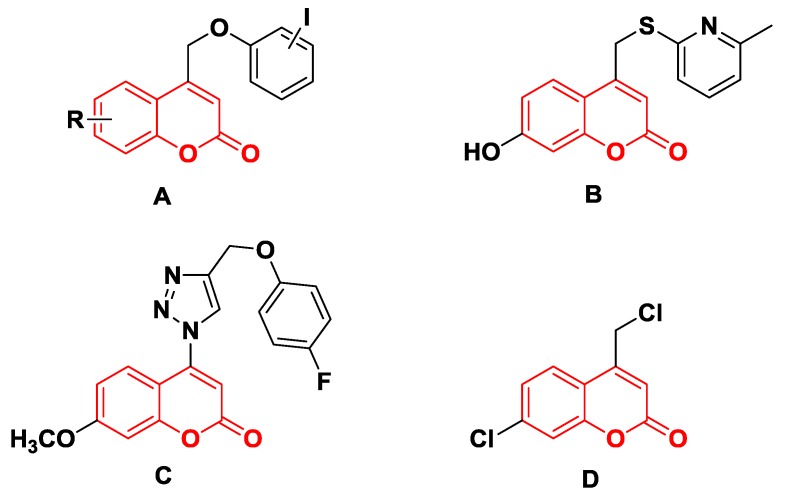
Structures of some 4-substituted coumarins. (**A**) the coumarins attached an iodinated aromatic ring. (**B**) the coumarin linked 6-methylpyridine. (**C**) the hybrid of 1,2,3-triazole and 4-subsitituted coumarin. (**D**) 4-substituted coumarins reported by supuran group.

For example, the coumarins attached an iodinated aromatic ring (Figure 1A), initially identified by Basanagouda et al. as a potential anti-cancer agent, exerted an anti-proliferative effect in MDA-MB-231 human adenocarcinoma mammary gland and A-549 human lung carcinoma [8]. Similarly, the coumarin linked 6-methylpyridine (Figure 1B) was reported to show potent inhibition of 17β-hydroxysteroid dehydrogenase type 3 (17β-HSD3) with an IC_50_ value of 0.0015 μM [9]. In addition, the hybrid of 1,2,3-triazole and 4-subsitituted coumarin (Figure 1C) had an IC_50_ value of 0.52 μM against A-549 cells and induces G2/M phase cell cycle arrest [10]. Interestingly, the supuran group revealed that 4-substituted coumarins (Figure 1D) are very effective against transmembrane, tumor-associated isoforms carbonic anhydrase IX (CA IX) [11] with activity in the submicromolar range [12]. From the precedents mentioned above, 4-substituted coumarin derivatives are thus excellent leads for designing antitumor agents.

**Figure 2:**
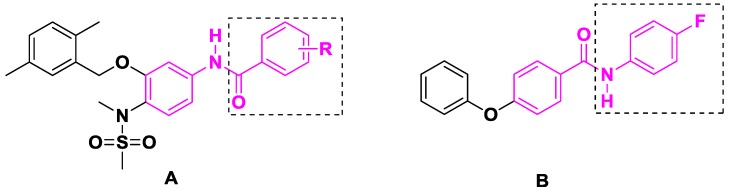
Structures of some benzanilide. (**A**) compounds reported by Su group. (**B**) compounds reported by Yang et al.

Benzanilide moieties are importantly active groups in anticancer agents and introduction of a benzanilide is considered as an efficient method to improve the activity of compounds. For instance, the Su group introduced electron-donating- or withdrawing group-substituted benzamides into compounds (Figure 2A) and some derivatives exhibited good growth inhibitory activity against SK-BR-3 breast cancer cells at low nanomolar concentrations [13]. Analogously, Yang et al. focused on the modification of the aniline in benzanilide, and identified the compound (Figure 2B) with an IC_50_ value of 2.57 μM, which inhibited HepG2 cell proliferation more effectively than sorafenib (IC_50_ = 9.61 μM) [14].

**Figure 3:**
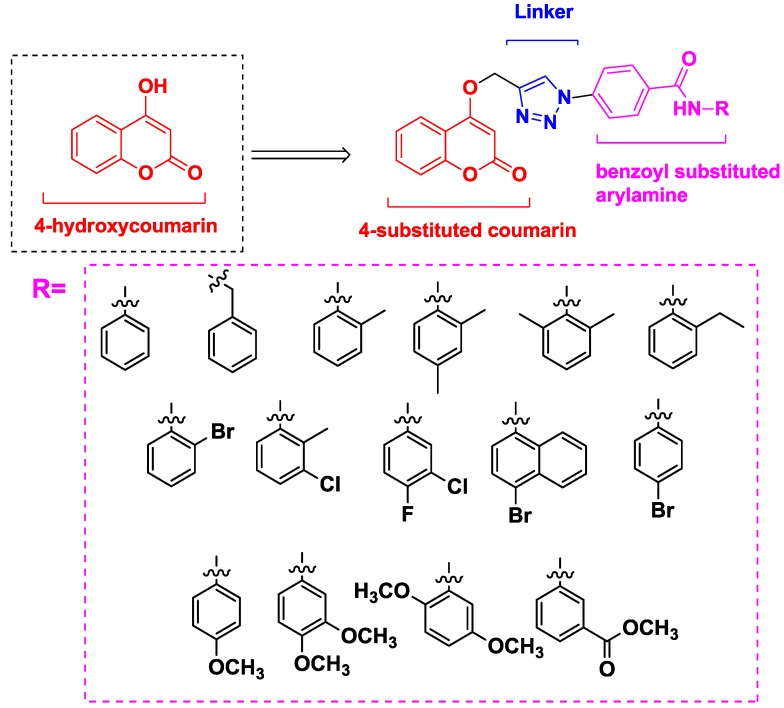
Rationale design of the title compounds.

Based on the molecular hybridization strategy and mentioned above, we combined a pharmacophore (coumarin) which can inhibit proliferation of cancer cells through varied mechanisms with another anticancer pharmacophore (benzanilide) which can increase the diversity of compounds to quickly screen target compounds with good anticancer activities. Herein, we referred to Pingaew’s work [15] and designed the compounds comprising three core structural elements (Figure 3): (i) a (4-substituted) coumarin moiety as a scaffold, (ii) a 1,2,3-triazole moiety as a biocompatible, covalent linker [16] and (iii) a benzoyl-substituted arylamine group as a variable group.

## 2. Results and Discussion

### 2.1. Chemistry

**Scheme 1:**
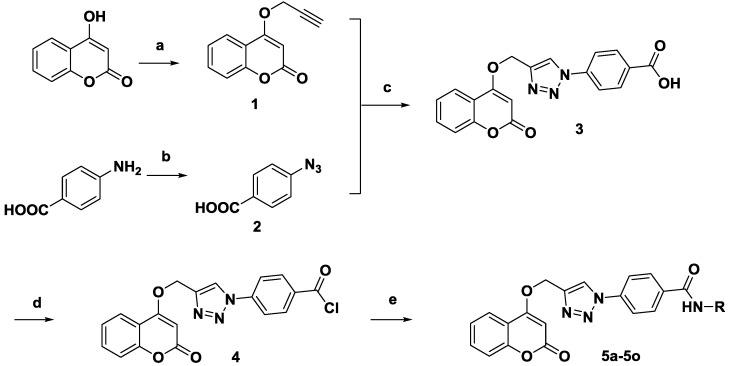
Synthesis of compounds **5a–5o**. *Reagents and conditions*: (**a**) Propargyl bromide, K_2_CO_3_, DMF, r.t., 4 h; (**b**) NaNO_2_, HCl, 0.5 h, NaN_3_, 0.5 h; (**c**) CuI, Et_3_N, DCM, r.t., 3 h; (**d**) SOCl_2_, 1.5 h; (**e**) RNH_2_, DCM, 2 h.

The synthesis of target compounds **5a**–**5o** was presented at Scheme 1. First, intermediate **1** was obtained by substitution of propargyl bromide in 4-hydroxycoumarin at room temperature [17], and **2** was synthesized by the azidation of 4-aminobenzoic acid at the presence of NaNO_2_/NaN_3_ in H_2_O. Next, **1** and **2** were treated with CuI in dichloromethane at room temperature via click chemistry to give compound **3** and then a chlorination reaction with SOCl_2_ was conducted to obtain intermediate **4**. Last, **4** was reacted with the corresponding substituted arylamines to obtain target compounds **5a**–**5o**.

### 2.2. Biological Evaluation

The antiproliferative activities of new compounds against MDA-MB-231 cells (a kind of breast cancer cells) was evaluated by the 3-(4,5-dimethyl-2-thiazolyl)-2,5-diphenyl-2-*H*-tetrazolium bromide (MTT) assay. Specifically, we aimed to identify compounds that inhibit proliferation more powerfully under hypoxic conditions, in line with the physiological response to hypoxia in increased CA IX activity in this cell line, so both normoxic and hypoxic conditions were evaluated [18]. Doxorubicin (DOX), cisplatin(*cis*-Pt) and 4-hydroxycoumarin were selected as positive reference drugs.

The in vitro antiproliferative activities are summarized in Table 1. From the structure-activity relationships of the target compounds the following was concluded:

(i) All compounds showed better inhibition than that of 4-hydroxycoumarin and **5e** had the best antiproliferative activity of this series of compounds, even better inhibition than that of DOX and *cis*-Pt under hypoxia;
(ii) The IC_50_ of most compounds under hypoxic conditions were lower than that under normoxic conditions;
(iii) The IC_50_, _normoxia_/IC_50_, _hypoxia_ of DOX and *cis*-Pt were lower than that of **5b**, **5e**, **5h**, **5m**, **5o**;
(iv) The presence of a 4-substitued phenyl in the R group enhances the antiproliferative potential and antiproliferative activity increases remarkably when a 3,4-substitued phenyl is present in the R group.

### 2.3. Molecular Docking

**Figure 4:**
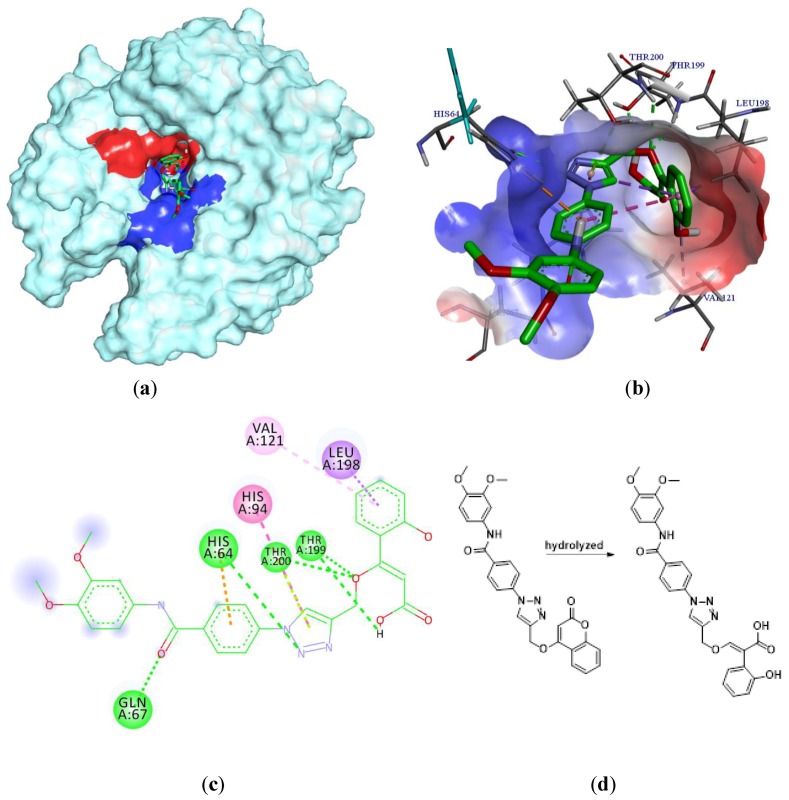
Interaction diagrams of the selected docked conformations for hydrolyzed compound **5e** inside the active site of CA IX enzyme. (**a**) The surface representation of binding pocket has been shown at the top of the figure. (**b**) 3D ligand interactions diagram. (**c**) 2D ligand interactions diagram. (**d**) The ligand of this docking analysis.

In the last years, several approaches have reported that the coumarins are truly CA IX-selective inhibitors [12,19,20]. The coumarin moiety is the scaffold of our newly synthesized compounds and we wanted to predict the binding mode of the most antiproliferative **5e** of this series of compounds into the binding site of carbonic anhydrase IX, so we decided to carry out a molecular docking study [21]. We knew that coumarin moiety was hydrolyzed within the CA IX active site [12,22]. Meanwhile, according to our previous work [23], as shown in Figure 4(d), the hydrolyzed compound **5e** was used as the ligand of this docking analysis. The molecular docking results (Figure 4(b,c)) showed that the carboxyl group in 2-hydroxycinnamic acid moiety engaged hydrogen bonds with the hydroxyl group of Thr199. The oxygen atom of enol ether acted as acceptor receiving two H-bonds from the backbone NH of Thr199 and Thr200. Meanwhile, the nitrogen atom of triazole moiety formed a H-bond with His64 and the carbonyl group of the benzoyl arylamine moiety formed H-bond with Gln67. In addition, the triazole moiety displayed a π-π stacking with His94, and the aromatic benzene ring of the coumarin group showed lipophilic interactions with Leu98 and Val121, respectively. As shown in Figure 4a,b, the coumarin moiety will generally adopt a conformation to interact with the hydrophobic half (red part) of the CA IX active site cavity; similarly, the triazole and the benzoyl aniline moieties will generally interact with the hydrophilic half (blue part). In addition, the binding energy is −6.57 kcal/mol. On the basis of the docking results, it was found that compound **5e** had the potential to inhibit CA IX, which will be the probable anticancer activity mechanism of these derivatives.

## 3. Materials and Methods

### 3.1. Chemistry

Column chromatography was carried out on the 200–300 mesh silica gel (Qingdao Haiyang Chemical Co. Ltd., Qingdao, Shandong, China). Analytical thin-layer chromatography (TLC) was performed on silica gel precoated GF254 plates (Qingdao Haiyang Chemical Co. Ltd.). ^1^H-NMR and ^13^C-NMR spectra were recorded on an AV-400 spectrometer (Bruker Bioscience, Billerica, MA, USA), with tetramethylsilane as an internal standard. ^1^H and ^13^C-NMR spectra of these compounds are available in the Supplementary Materials. ESI-MS spectra were obtained on an Agilent ESI-QTOF instrument. High resolution mass spectra (HRMS) were measured with an Agilent Accurate-Mass Q-TOF 6530 (Agilent, Santa Clara, CA, USA) in ESI mode and are available in the Supplementary Materials. Melting points were determined using a X-4 microscope melting point apparatus (Beijing Tech Instrument Co., Ltd., Beijing, China) without calibration.

#### 3.1.1. 4-(Prop-2-ynyloxy)-2*H*-chromen-2-one (**1**)

4-Hydroxycoumarin (20 g, 0.12 mol) was dissolved in DMF (100 mL), and K_2_CO_3_ (2 eq) was added. Propargyl bromide (1.5 eq) was then added under nitrogen. The reaction mixture was kept stirring at room temperature. After the completion of reaction (as monitored by TLC), the reaction mixture was poured onto crushed ice and set aside for some time. Then it was filtered and dried to obtain the desired product. The crude product was used without further purification. Yield 63%; m.p. 155–156 °C ^1^H-NMR (DMSO-*d*_6_, 600 MHz, δ, TMS = 0): 7.82–7.75 (m, 1H), 7.71–7.64 (m, 1H), 7.44–7.39 (m, 1H), 7.40–7.34 (m, 1H), 5.96 (d, *J* = 1.3 Hz, 1H), 5.11 (dd, *J* = 2.4, 1.1 Hz, 2H), 3.83 (t, *J* = 2.4 Hz, 1H). ESI-MS [M − H]^−^: (*m*/*z*) Calcd. for C_12_H_7_O_3_: 199.0. Found: 199.1.

#### 3.1.2. 4-Azidobenzoic acid (**2**)

4-Aminobenzoic acid (11.2 g, 0.08 mol) and 3 M HCl (250 mL) were added to a 500 mL three-necked flask, and then cooled to 0 °C. NaNO_2_ (aq, 6.8g (0.098 mol)/50 mL) was added dropwise to the cooled mixture while the temperature was kept between 0 and 5 °C. After stirring for 30 min, NaN_3_ (aq, 7.6g (0.12 mol)/50 mL) was added dropwise to the cooled mixture. The reaction mixture was stirred for 30 min at 0 °C and 1 h at room temperature. Then it was filtered, washed with water and dried to obtain the desired product. The crude product was used without further purification. Yield 90%; m.p. 178–180 °C. ^1^H-NMR (DMSO-*d*_6_, 600 MHz, δ, TMS = 0):12.97 (s, 1H), 8.01–7.90 (m, 2H), 7.26–7.15 (m, 2H). ESI-MS [M − H]^−^: (*m*/*z*) Calcd. for C_7_H_4_N_3_O_2_: 162.0 Found: 162.0.

#### 3.1.3. 4-(4-(((2-Oxo-2*H*-chromen-4-yl)oxy)methyl)-1*H*-1,2,3-triazol-1-yl)benzoic acid (**3**)

In a 250 mL flask, compounds **1** (10 g, 0.05 mol) and **2** (9 g, 0.055 mol) were added to DCM (150 mL) at room temperature. To this mixture was added CuI 1g (5 mmol), followed by trimethylamine 2 g (0.02 mol) under an argon atmosphere. After the completion of reaction (as evidenced by TLC), the mixture was washed with 1 M HCl and evaporated. The residue was directly used in the next step without further purification. Yield 90%; m.p. 218–220 °C. ^1^H-NMR (DMSO-*d*_6_, 600 MHz, δ, TMS = 0): 9.18 (s, 1H), 8.15 (d, *J* = 8.2 Hz, 2H), 8.07 (d, *J* = 8.2 Hz, 2H), 7.84 (d, *J* = 7.9 Hz, 1H), 7.66 (t, *J* = 7.7 Hz, 1H), 7.42 (d, *J* = 8.3 Hz, 1H), 7.35 (t, *J* = 7.6 Hz, 1H), 6.21 (s, 1H), 5.54 (s, 2H). ESI-MS [M − H]^−^: (*m*/*z*) Calcd. for C_19_H_12_N_3_O_5_: 362.1 Found: 362.1.

#### 3.1.4. Synthesis of Compound **4**

In a 100 mL flask, compound **3** (1 g, 2.8 mmol) were added to anhydrous DCM (50 mL), and cooled to 0 °C. Then, sulfoxide chloride (15 mL) was added dropwise to the cooled mixture under stirring. After added, the reaction mixture was stirred for 2 h at room temperature. Solvent and excess sulfoxide chloride were evaporated. The residue was directly used in the next step without further purification.

#### 3.1.5. General Procedure for the Synthesis of Compound **5a**–**5e**

In a 50 mL flask, the appropriate arylamine (1 mmol) was added to anhydrous DCM (10 mL), and cooled to 0 °C. Then, compound **4** (0.2 g, 0.5 mmol) mixed with anhydrous DCM (4 mL) was added dropwise to the cooled mixture under stirring. After stirring for 30 min, the reaction mixture was stirred for 4 h more at room temperature. After the completion of reaction (as evidenced by TLC), the mixture was evaporated. The residue was finally purified by column chromatography (DCM:MeOH = 50:1) to obtain the desired products.

*4-(4-(((2-Oxo-2H-chromen-4-yl)oxy)methyl)-1H-1,2,3-triazol-1-yl)-N-arylbenzamide* (**5a**): Yield 78%, m.p. 239–42 °C. ^1^H-NMR (DMSO-*d*_6_, 600 MHz, δ, TMS = 0): 10.40 (s, 1H), 9.22 (s, 1H), 8.21 (d, *J* = 8.6 Hz, 2H), 8.15 (d, *J* = 8.7 Hz, 2H), 7.85 (dd, *J* = 8.0, 1.6 Hz, 1H), 7.80 (d, *J* = 7.3 Hz, 2H), 7.67 (ddd, *J* = 8.6, 7.3, 1.7 Hz, 1H), 7.42 (dd, *J* = 8.3, 1.0 Hz, 1H), 7.40–33 (m, 3H), 7.13 (tt, *J* = 7.3, 1.2 Hz, 1H), 6.23 (s, 1H), 5.56 (s, 2H). ^13^C-NMR (DMSO-*d*_6_, 150 MHz, δ, TMS = 0): 164.46, 161.67, 152.91, 142.68, 139.07, 138.59, 135.01, 133.01, 129.63, 128.79, 124.37, 124.02, 123.63, 123.17, 120.58, 119.95, 116.60, 115.16, 91.63, 62.90. ESI-HRMS [M − H]^−^: (*m*/*z*) Calcd. for C_25_H_17_N_4_O_4_: 437.1250. Found: 437.1271.

*4-(4-(((2-Oxo-2H-chromen-4-yl)oxy)methyl)-1H-1,2,3-triazol-1-yl)-N-(o-tolyl)benzamide* (**5b**): Yield 72%, m.p. 248–250 °C. ^1^H-NMR (DMSO-*d*_6_, 600 MHz, δ, TMS = 0): 10.06 (s, 1H), 9.22 (s, 1H), 8.22 (d, *J* = 8.5 Hz, 2H), 8.15 (d, *J* = 8.7 Hz, 2H), 7.86 (dd, *J* = 8.0, 1.6 Hz, 1H), 7.67 (ddd, *J* = 8.6, 7.3, 1.6 Hz, 1H), 7.43 (dd, *J* = 8.3, 1.0 Hz, 1H), 7.39–7.34 (m, 2H), 7.32–7.27 (m, 1H), 7.24 (td, *J* = 7.6, 1.7 Hz, 1H), 7.19 (td, *J* = 7.4, 1.4 Hz, 1H), 6.23 (s, 1H), 5.56 (s, 2H), 2.26 (s, 3H). ^13^C-NMR (DMSO-*d*_6_, 150 MHz, δ, TMS = 0): 164.44, 164.25, 161.65, 152.89, 142.68, 138.58, 136.29, 134.58, 133.93, 132.99, 130.48, 129.56, 126.78, 126.30, 126.17, 124.35, 123.60, 123.16, 119.97, 116.58, 115.15, 91.62, 62.89, 18.03. ESI-HRMS [M − H]^−^: (*m*/*z*) Calcd. for C_26_H_19_N_4_O_4_: 451.1406. Found: 451.1415.

*N-(2-Bromophenyl)-4-(4-(((2-oxo-2H-chromen-4-yl)oxy)methyl)-1H-1,2,3-triazol-1-yl)benzamide* (**5c**): Yield 55%, m.p. 285–287 °C. ^1^H-NMR (DMSO-*d*_6_, 600 MHz, δ, TMS = 0): 10.54 (s, 1H), 9.22 (s, 1H), 8.20 (d, *J* = 8.4 Hz, 2H), 8.16 (d, *J* = 8.4 Hz, 2H), 8.13 (d, *J* = 2.3 Hz, 1H), 7.88–7.82 (m, 1H), 7.78 (d, *J* = 7.8 Hz, 1H), 7.67 (t, *J* = 7.9 Hz, 1H), 7.43 (d, *J* = 8.3 Hz, 1H), 7.34 (dt, *J* = 21.7, 7.6 Hz, 3H), 6.22 (s, 1H), 5.56 (s, 2H). ^13^C-NMR (DMSO-*d*_6_, 150 MHz, δ, TMS = 0): 164.48, 164.39, 161.70, 152.92, 142.73, 138.82, 136.44, 134.07, 133.04, 132.88, 129.64, 129.28, 128.38, 128.32, 124.39, 123.65, 123.19, 120.96, 120.11, 116.62, 115.17, 91.64, 62.91. ESI-HRMS [M − H]^−^: (*m*/*z*) Calcd. for C_25_H_16_N_4_O_4_Br: 517.0334. Found: 517.0327.

*N-(3-Bromophenyl)-4-(4-(((2-oxo-2H-chromen-4-yl)oxy)methyl)-1H-1,2,3-triazol-1-yl)benzamide* (**5d**): Yield 58%, m.p. 289–292 °C. ^1^H-NMR (DMSO-*d*_6_, 600 MHz, δ, TMS = 0): 10.56 (s, 1H), 9.23 (s, 1H), 8.21 (d, *J* = 8.7 Hz, 2H), 8.16 (d, *J* = 8.7 Hz, 2H), 8.13 (d, *J* = 2.1 Hz, 1H), 7.86 (dd, *J* = 7.9, 1.6 Hz, 1H), 7.79 (dt, *J* = 7.9, 1.6 Hz, 1H), 7.68 (ddd, *J* = 8.7, 7.3, 1.6 Hz, 1H), 7.43 (d, *J* = 8.3 Hz, 1H), 7.40–7.30 (m, 3H), 6.23 (s, 1H), 5.56 (s, 2H). ^13^C-NMR (DMSO-*d*_6_, 150 MHz, δ, TMS = 0): 164.62, 164.38, 161.59, 152.82, 142.62, 140.62, 138.69, 134.48, 132.94, 130.75, 129.63, 126.52, 124.30, 123.57, 123.09, 122.70, 121.47, 119.92, 119.15, 116.53, 115.07, 91.55, 62.81. ESI-HRMS [M − H]^−^: (*m*/*z*) Calcd. for C_25_H_16_N_4_O_4_Br: 517.0334. Found: 517.0327.

*N-(3,4-Dimethoxyphenyl)-4-(4-(((2-oxo-2H-chromen-4-yl)oxy)methyl)-1H-1,2,3-triazol-1-yl)benzamide* (**5e**): Yield 80%; m.p. 283–286 °C. ^1^H-NMR (DMSO-*d*_6_, 600 MHz, δ, TMS = 0): 10.26 (s, 1H), 9.21 (s, 1H), 8.20 (d, 2H), 8.14 (d, 2H), 7.85 (dd, *J* = 8.0, 1.6 Hz, 1H), 7.67 (ddd, *J* = 8.6, 7.3, 1.7 Hz, 1H), 7.49 (d, *J* = 2.4 Hz, 1H), 7.42 (dd, *J* = 8.4, 1.0 Hz, 1H), 7.36 (td, *J* = 8.4, 1.9 Hz, 2H), 6.95 (d, *J* = 8.7 Hz, 1H), 6.22 (s, 1H), 5.56 (s, 2H), 3.77 (s, 3H), 3.75 (s, 3H). ^13^C-NMR (DMSO-*d*_6_, 150 MHz, δ, TMS = 0): 164.45, 163.96, 161.65, 152.89, 148.52, 145.40, 142.66, 138.48, 135.06, 132.54, 129.46, 124.36, 123.61, 123.16, 119.93, 116.59, 115.15, 112.55, 111.93, 105.62, 91.62, 62.90, 55.80, 55.51. ESI-HRMS [M − H]^−^: (*m*/*z*) Calcd. for C_27_H_21_N_4_O_6_: 497.1461. Found: 497.1475.

*N-(3-Chloro-2-methylphenyl)-4-(4-(((2-oxo-2H-chromen-4-yl)oxy)methyl)-1H-1,2,3-triazol-1-yl)benzamide* (**5f**): Yield 61%; m.p. 270–273 °C. ^1^H-NMR (DMSO-*d*_6_, 600 MHz, δ, TMS = 0): 10.31 (s, 1H), 9.22 (s, 1H), 8.23 (d, *J* = 8.6 Hz, 2H), 8.16 (d, *J* = 8.7 Hz, 2H), 7.85 (dd, *J* = 7.9, 1.6 Hz, 1H), 7.67 (ddd, *J* = 8.6, 7.3, 1.6 Hz, 1H), 7.43 (dd, *J* = 8.3, 1.0 Hz, 1H), 7.39 (dd, *J* = 8.0, 1.3 Hz, 1H), 7.38–7.33 (m, 2H), 7.28 (t, *J* = 7.9 Hz, 1H), 6.23 (s, 1H), 5.56 (s, 2H), 2.27 (s, 3H). ^13^C-NMR (DMSO-*d*_6_, 150 MHz, δ, TMS = 0): 164.49, 164.45, 161.65, 152.90, 142.70, 138.73, 137.95, 134.17, 133.95, 133.00, 132.40, 129.65, 127.07, 126.06, 124.35, 123.61, 123.16, 120.00, 116.59, 115.15, 91.62, 62.89, 15.50. ESI-HRMS [M − H]^−^: (*m*/*z*) Calcd. for C_26_H_18_N_4_O_4_Cl: 485.1017. Found: 485.1016.

*N-(2,5-Dimethoxyphenyl)-4-(4-(((2-oxo-2H-chromen-4-yl)oxy)methyl)-1H-1,2,3-triazol-1-yl)benzamide* (**5g**): Yield 74%; m.p. 267–269 °C. ^1^H-NMR (DMSO-*d*_6_, 600 MHz, δ, TMS = 0): 9.61 (s, 1H), 9.21 (s, 1H), 8.19 (d, *J* = 8.2 Hz, 2H), 8.13 (d, *J* = 8.3 Hz, 2H), 7.85 (d, *J* = 7.9 Hz, 1H), 7.67 (t, *J* = 7.9 Hz, 1H), 7.49 (d, *J* = 3.1 Hz, 1H), 7.43 (d, *J* = 8.3 Hz, 1H), 7.36 (t, *J* = 7.6 Hz, 1H), 7.03 (d, *J* = 8.9 Hz, 1H), 6.77 (dd, *J* = 9.0, 3.2 Hz, 1H), 6.22 (s, 1H), 5.56 (s, 2H), 3.80 (s, 3H), 3.73 (s, 3H). ^13^C-NMR (DMSO-*d*_6_, 150 MHz, δ, TMS = 0): 164.43, 164.03, 161.64, 152.92, 152.88, 145.71, 142.68, 138.62, 134.52, 132.98, 129.48, 127.44, 124.34, 123.57, 123.16, 120.00, 116.57, 115.14, 112.27, 110.77, 110.07, 91.61, 62.89, 56.32, 55.54. ESI-HRMS [M − H]^−^: (*m*/*z*) Calcd. for C_27_H_21_N_4_O_6_: 497.1461. Found: 497.1473.

*N-(2-Ethylphenyl)-4-(4-(((2-oxo-2H-chromen-4-yl)oxy)methyl)-1H-1,2,3-triazol-1-yl)benzamide* (**5h**): Yield 70%; m.p. 244–246 °C. ^1^H-NMR (DMSO-*d*_6_, 600 MHz, δ, TMS = 0): 10.07 (s, 1H), 9.21 (s, 1H), 8.22 (d, *J* = 8.6 Hz, 2H), 8.15 (d, *J* = 8.6 Hz, 2H), 7.86 (dd, *J* = 8.0, 1.6 Hz, 1H), 7.67 (ddd, *J* = 8.6, 7.3, 1.6 Hz, 1H), 7.43 (dd, *J* = 8.3, 1.0 Hz, 1H), 7.36 (ddd, *J* = 8.1, 7.3, 1.1 Hz, 1H), 7.34–7.29 (m, 2H), 7.29–7.22 (m, 2H), 6.23 (s, 1H), 5.56 (s, 2H), 2.65 (q, *J* = 7.6 Hz, 2H), 1.15 (t, *J* = 7.6 Hz, 3H). ^13^C-NMR (DMSO-*d*_6_, 150 MHz, δ, TMS = 0): 164.65, 164.45, 161.65, 152.89, 142.68, 140.02, 138.59, 135.65, 134.56, 132.98, 129.51, 128.66, 127.74, 126.81, 126.20, 124.33, 123.59, 123.15, 120.01, 116.57, 115.14, 91.62, 62.89, 24.10, 14.28. ESI-HRMS [M − H]^−^: (*m*/*z*) Calcd. for C_27_H_21_N_4_O_4_: 465.1563. Found: 465.1567.

*N-(4-Methoxyphenyl)-4-(4-(((2-oxo-2H-chromen-4-yl)oxy)methyl)-1H-1,2,3-triazol-1-yl)benzamide* (**5i**): Yield 70%; m.p. 283–287 °C. ^1^H-NMR (DMSO-*d*_6_, 600 MHz, δ, TMS = 0): 10.32 (s, 1H), 9.22 (s, 1H), 8.20 (d, *J* = 8.4 Hz, 2H), 8.14 (d, *J* = 8.4 Hz, 2H), 7.85 (dd, *J* = 8.0, 1.6 Hz, 1H), 7.68 (d, *J* = 6.1 Hz, 2H), 7.43 (d, *J* = 8.3 Hz, 1H), 7.36 (t, *J* = 7.6 Hz, 1H), 7.18 (d, *J* = 8.2 Hz, 2H), 6.75 (d, *J* = 8.6 Hz, 1H), 6.23 (s, 1H), 5.56 (s, 2H), 3.32 (s, 3H). ^13^C-NMR (DMSO-*d*_6_, 150 MHz, δ, TMS = 0): 164.37, 163.89, 161.57, 155.71, 152.81, 142.58, 138.38, 134.99, 132.92, 132.01, 129.41, 124.28, 123.52, 123.08, 122.10, 119.84, 116.51, 115.07, 113.81, 91.54, 62.81, 55.22. ESI-HRMS [M − H]^−^: (*m*/*z*) Calcd. for C_26_H_19_N_4_O_5_: 467.1355. Found: 467.1366.

*N-(2,6-Dimethylphenyl)-4-(4-(((2-oxo-2H-chromen-4-yl)oxy)methyl)-1H-1,2,3-triazol-1-yl)benzamide* (**5j**): Yield 68%; m.p. 281–284 °C. ^1^H-NMR (DMSO-*d*_6_, 600 MHz, δ, TMS = 0): 9.95 (s, 1H), 9.21 (s, 1H), 8.24 (d, *J* = 8.7 Hz, 2H), 8.15 (d, *J* = 8.7 Hz, 2H), 7.86 (dd, *J* = 7.9, 1.6 Hz, 1H), 7.67 (ddd, *J* = 8.6, 7.3, 1.6 Hz, 1H), 7.43 (dd, *J* = 8.4, 1.0 Hz, 1H), 7.36 (ddd, *J* = 8.1, 7.3, 1.1 Hz, 1H), 7.14 (s, 3H), 6.23 (s, 1H), 5.56 (s, 2H), 2.21 (s, 6H). ^13^C-NMR (DMSO-*d*_6_, 150 MHz, δ, TMS = 0): 164.42, 163.93, 161.62, 152.88, 142.65, 138.58, 135.69, 135.16, 134.40, 132.96, 129.39, 127.87, 126.91, 124.32, 123.60, 123.14, 120.06, 116.56, 115.13, 91.61, 62.89, 18.17. ESI-HRMS [M − H]^−^: (*m*/*z*) Calcd. for C_27_H_21_N_4_O_4_: 465.1563. Found: 465.1563.

*N-(2,4-Dimethylphenyl)-4-(4-(((2-oxo-2H-chromen-4-yl)oxy)methyl)-1H-1,2,3-triazol-1-yl)benzamide* (**5k**): Yield 71%; m.p. 270–273 °C. ^1^H-NMR (DMSO-*d*_6_, 600 MHz, δ, TMS = 0): 1H-NMR (600 MHz, DMSO-*d*_6_) δ 9.98 (s, 1H), 9.21 (s, 1H), 8.21 (d, *J* = 8.3 Hz, 2H), 8.13 (d, *J* = 8.7 Hz, 2H), 7.86 (dd, *J* = 7.9, 1.6 Hz, 1H), 7.67 (ddd, *J* = 8.6, 7.3, 1.7 Hz, 1H), 7.43 (dd, *J* = 8.4, 1.0 Hz, 1H), 7.36 (ddd, *J* = 8.2, 7.3, 1.1 Hz, 1H), 7.22 (d, *J* = 7.9 Hz, 1H), 7.14–7.07 (m, 1H), 7.06–7.00 (m, 1H), 6.23 (s, 1H), 5.56 (s, 2H), 2.29 (s, 3H), 2.21 (s, 3H). ^13^C-NMR (DMSO-*d*_6_, 150 MHz, δ, TMS = 0): 164.47, 164.26, 161.67, 152.91, 142.68, 138.54, 135.43, 134.66, 133.75, 133.68, 133.01, 131.01, 129.53, 126.73, 126.70, 124.37, 123.60, 123.17, 119.97, 116.60, 115.16, 91.63, 62.90, 20.68, 17.96. ESI-HRMS [M − H]^−^: (*m*/*z*) Calcd. for C_27_H_21_N_4_O_4_: 465.1563. Found: 465.1568.

*N-(3-Chloro-4-fluorophenyl)-4-(4-(((2-oxo-2H-chromen-4-yl)oxy)methyl)-1H-1,2,3-triazol-1-yl)benzamide* (**5l**): Yield 73%; m.p. 277–279 °C. ^1^H-NMR (DMSO-*d*_6_, 600 MHz, δ, TMS = 0): 10.58 (s, 1H), 9.22 (s, 1H), 8.20 (d, *J* = 8.8 Hz, 2H), 8.16 (d, *J* = 8.8 Hz, 2H), 8.10 (dd, *J* = 6.9, 2.6 Hz, 1H), 7.85 (dd, *J* = 7.9, 1.6 Hz, 1H), 7.75 (ddd, *J* = 9.0, 4.3, 2.6 Hz, 1H), 7.67 (ddd, *J* = 8.6, 7.3, 1.7 Hz, 1H), 7.48–7.40 (m, 2H), 7.36 (ddd, *J* = 8.2, 7.3, 1.1 Hz, 1H), 6.22 (s, 1H), 5.56 (s, 2H). ^13^C-NMR (DMSO-*d*_6_, 150 MHz, δ, TMS = 0): 164.50, 164.35, 161.56, 152.81, 142.61, 138.70, 136.23, 134.33, 132.92, 129.58, 124.27, 123.54, 123.06, 121.87, 120.79, 120.74, 119.92, 117.00, 116.86, 116.51, 115.06, 91.54, 62.80. ESI-HRMS [M − H]^−^: (*m*/*z*) Calcd. for C_25_H_15_N_4_O_4_FCl: 489.0766. Found: 489.0780.

*Methyl 3-(4-(4-(((2-oxo-2H-chromen-4-yl)oxy)methyl)-1H-1,2,3-triazol-1-yl)benzamido)benzoate* (**5m**): Yield 55%; m.p. 256–258 °C. ^1^H-NMR (DMSO-*d*_6_, 600 MHz, δ, TMS = 0):10.62 (s, 1H), 9.23 (s, 1H), 8.48 (t, *J* = 2.0 Hz, 1H), 8.24 (d, *J* = 8.7 Hz, 2H), 8.16 (d, *J* = 8.7 Hz, 2H), 8.11 (ddd, *J* = 8.2, 2.3, 1.1 Hz, 1H), 7.85 (dd, *J* = 8.0, 1.6 Hz, 1H), 7.72 (dt, *J* = 7.7, 1.4 Hz, 1H), 7.67 (ddd, *J* = 8.6, 7.3, 1.6 Hz, 1H), 7.54 (t, *J* = 7.9 Hz, 1H), 7.43 (dd, *J* = 8.3, 1.1 Hz, 1H), 7.36 (ddd, *J* = 8.2, 7.3, 1.1 Hz, 1H), 6.23 (s, 1H), 5.56 (s, 2H), 3.88 (s, 3H). ^13^C-NMR (DMSO-*d*_6_, 150 MHz, δ, TMS = 0): 166.11, 164.53, 164.35, 161.56, 152.81, 142.60, 139.41, 138.65, 134.48, 132.91, 130.08, 129.61, 129.22, 124.84, 124.47, 124.27, 123.54, 123.07, 120.91, 119.89, 116.50, 115.06, 91.54, 62.81, 52.27. ESI-HRMS [M − H]^−^: (*m*/*z*) Calcd. for C_27_H_19_N_4_O_6_: 495.1305. Found: 495.1327.

*N-Benzyl-4-(4-(((2-oxo-2H-chromen-4-yl)oxy)methyl)-1H-1,2,3-triazol-1-yl)benzamide* (**5n**): Yield 60%; m.p. 249–251 °C. ^1^H-NMR (DMSO-*d*_6_, 600 MHz, δ, TMS = 0): 9.22 (t, *J* = 6.0 Hz, 1H), 9.19 (s, 1H), 8.14 (d, *J* = 8.8 Hz, 2H), 8.09 (d, *J* = 8.7 Hz, 2H), 7.84 (dd, *J* = 7.9, 1.6 Hz, 1H), 7.67 (ddd, *J* = 8.6, 7.3, 1.6 Hz, 1H), 7.42 (dd, *J* = 8.3, 1.0 Hz, 1H), 7.37–7.32 (m, 5H), 7.25 (tt, *J* = 5.8, 3.0 Hz, 1H), 6.22 (s, 1H), 5.55 (s, 2H), 4.52 (d, *J* = 5.9 Hz, 2H). ^13^C-NMR (DMSO-*d*_6_, 150 MHz, δ, TMS = 0): 165.15, 164.42, 161.63, 152.88, 142.63, 139.56, 138.39, 134.39, 132.95, 129.14, 128.42, 127.37, 126.91, 124.31, 123.53, 123.14, 119.92, 116.55, 115.13, 91.60, 62.89, 42.83. ESI-HRMS [M − H]^−^: (m/z) Calcd. for C_26_H_19_N_4_O_4_: 451.1406. Found: 451.1404.

*N-(4-Bromonaphthalen-1-yl)-4-(4-(((2-oxo-2H-chromen-4-yl)oxy)methyl)-1H-1,2,3-triazol-1-yl)benzamide* (**5o**): Yield 65%; m.p. 306–309 °C. ^1^H-NMR (DMSO-*d*_6_, 600 MHz, δ, TMS = 0):10.69 (s, 1H), 9.25 (s, 1H), 8.33 (d, *J* = 8.2 Hz, 2H), 8.20 (t, *J* = 7.9 Hz, 3H), 8.12 (d, *J* = 8.5 Hz, 1H), 7.96 (d, *J* = 7.9 Hz, 1H), 7.86 (d, *J* = 7.9 Hz, 1H), 7.75 (t, *J* = 7.6 Hz, 1H), 7.68 (q, *J* = 7.1 Hz, 2H), 7.60 (d, *J* = 7.9 Hz, 1H), 7.43 (d, *J* = 8.3 Hz, 1H), 7.37 (t, *J* = 7.6 Hz, 1H), 6.24 (s, 1H), 5.57 (s, 2H). ^13^C-NMR (DMSO-*d*_6_, 150 MHz, δ, TMS = 0): 165.15, 164.36, 161.57, 152.81, 142.63, 138.69, 134.19, 134.02, 132.92, 131.61, 130.42, 129.74, 129.71, 128.10, 127.13, 126.74, 124.70, 124.28, 124.26, 123.54, 123.08, 119.91, 119.51, 116.51, 115.07, 91.55, 62.82. ESI-HRMS [M − H]^−^: (*m*/*z*) Calcd. for C_29_H_18_N_4_O_4_Br: 567.0491. Found: 567.0511.

### 3.2. In-Vitro Cytotoxicity Study (MTT Assay)

The cells suspended in the corresponding culture medium were inoculated in 96-well microtiter plates at a density of 1500–3000 cells per well, and incubated for 24 h at 37 °C in a humidified atmosphere with 95% air and 5% CO_2_. Different concentrations (100, 50, 25, 12.5 and 6.25 μM) of DOX, *cis*-Pt or the test compounds were added in the line of cell (MDA-MB-231) in 96 h incubation. The cells were incubated for 4 h with 20 μL of 5 mg/mL MTT solution. Supernatant from each well was carefully removed, and the media were then replaced with 100 μL of dimethyl sulfoxide to dissolve the purple colored formazan crystals formed in the wells, and their absorbance were measured at 492 nm with a microplate reader (Synergy-HT, BioTek Instruments, Winooski, VT, USA); 100 μL DMSO was set as the blank control. The hypoxic condition was achieved by placing cells in a sealed hypoxia incubator chamber (Catalog Number 27310, Stemcell Technologies, Inc., Vancouver, BC, Canada) filled with 5% CO_2_ and 95% N_2_ [23]. For the hypoxia group, Different concentrations (100, 50, 25, 12.5 and 6.25 μM) of DOX, *cis*-Pt or the test compounds were added in the line of cell (MDA-MB-231) in 24 h incubation under hypoxia condition. Then the cells were moved into normoxic condition and cultured for additional 72 h.

### 3.3. Molecular Docking Simulations

The experimental crystallographic structures of CA IX complex were from the Protein DataBank (PDB ID: 4ZAO). Both the protein and the ligand were prepared by adding partial charges and polar hydrogen atoms with the assistance of AutoDock Tools (version 1.5.6, The Scripps Research Institute, San Diego, CA, USA). A grid box size of 62.0, 66.0, 66.0 Å was generated to cover the active pocket of the receptor. We set the energy evaluations as the maximum of 2.5 × 10^6^ in the grid point spacing of 0.375 Å and performed 200 independent runs of genetic algorithm. All other parameters were set to default unless stated otherwise. Two-dimensional and Three-dimensional schematic representation of protein-ligand interaction was generated using Discovery Studio 2016 client (v16.1.0.15350, Dassault Systemes Biovia Company, Boston, MA. USA).

## 4. Conclusions

In conclusion, a series of compounds containing a 4-substituted coumarin moiety were designed, synthesized and evaluated for their antitumor activities. These compounds exhibited moderate antiproliferative activities against the MDA-MB-231 cell line under both normoxic and hypoxic conditions, and all displayed better activity than the parent compound 4-hydroxycoumarin. Furthermore, compound **5e** showed the most prominent anticancer activities of this series of compounds with about 5000-fold more activity than 4-hydroxycoumarin and 20-fold more than doxorubicin. Meanwhile, almost all of the compounds revealed a general promotion of proliferation inhibiting activity under hypoxia, contrasted with normoxia. Molecular docking simulations were performed to elucidate the mode(s) of binding for compound **5e**. The results showed compound that **5e** had potential to inhibit CA IX. Further studies on the mechanism of these compounds’ anticancer activity are underway.

## Figures and Tables

**Table 1:**
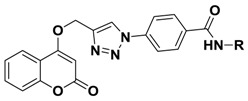
IC_50_ of the compounds against MDA-MB-231 cell.

Compd.	R	IC_50_ (Μm) ^a^		IC_50, normoxia_/IC_50, hypoxia_^b^
		Hypoxia	Normoxic	
**5a**	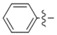	23.47	24.41	1.04
**5b**	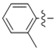	8.14	108.72	13.36
**5c**	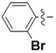	75.21	73.77	0.98
**5d**	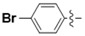	6.72	6.78	1.01
**5e**	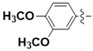	0.03	1.34	46.31
**5f**	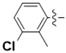	73.82	91.61	1.24
**5g**	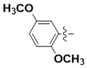	53.98	62.79	1.16
**5h**	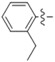	3.44	18.45	5.36
**5i**	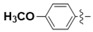	20.35	20.98	1.03
**5j**	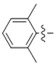	12.87	12.28	0.95
**5k**	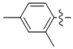	8.70	10.86	1.25
**5l**	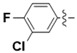	1.30	7.03	5.39
**5m**	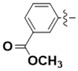	0.25	5.06	20.46
**5n**	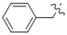	34.82	39.58	1.14
**5o**	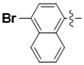	9.42	16.76	1.78
DOX		0.60	1.07	1.79
*cis*-Pt		4.68	7.87	1.68
4-hydroxycoumarin		>100	>100	

^a^ Values are the average of three independent experiments. Relative errors are generally within 5–10%. ^b^ A higher IC_50, normoxia_/IC_50, hypoxia_ indicates more antiproliferative activity in hypoxic conditions.

## Supplementary Materials

^1^H and ^13^C-NMR spectra of these compounds are available in the supplementary materials.
