# Supplementary material for: Design, Synthesis and Biological Evaluation of Novel 4-Substituted Coumarin Derivatives as Antitumor Agents
Source: Molecules. 2018 Sep 6;23(9):2281. doi: 10.3390/molecules23092281 (PMC6225359; doi:10.3390/molecules23092281)
Supplement: Supplementary file 1 [file molecules-23-02281-s001.pdf]

Characterization data:

5a

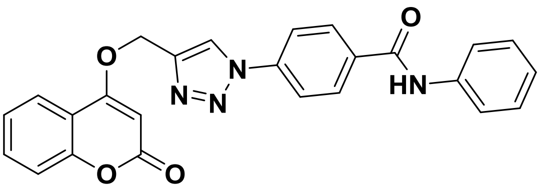

4-(4-(((2-oxo-2*H*-chromen-4-yl)oxy)methyl)-1*H*-1,2,3-triazol-1-yl)-*N*-phenylbenzamide

HRMS of 5a

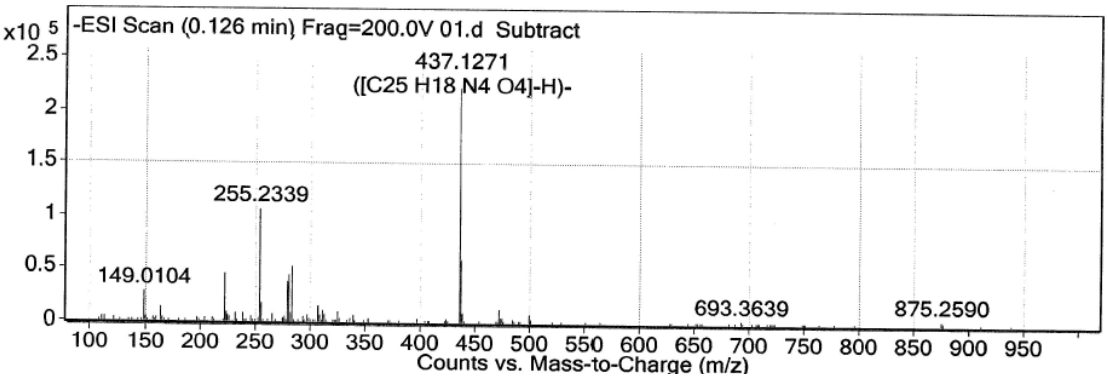

^{1}H NMR of 5a

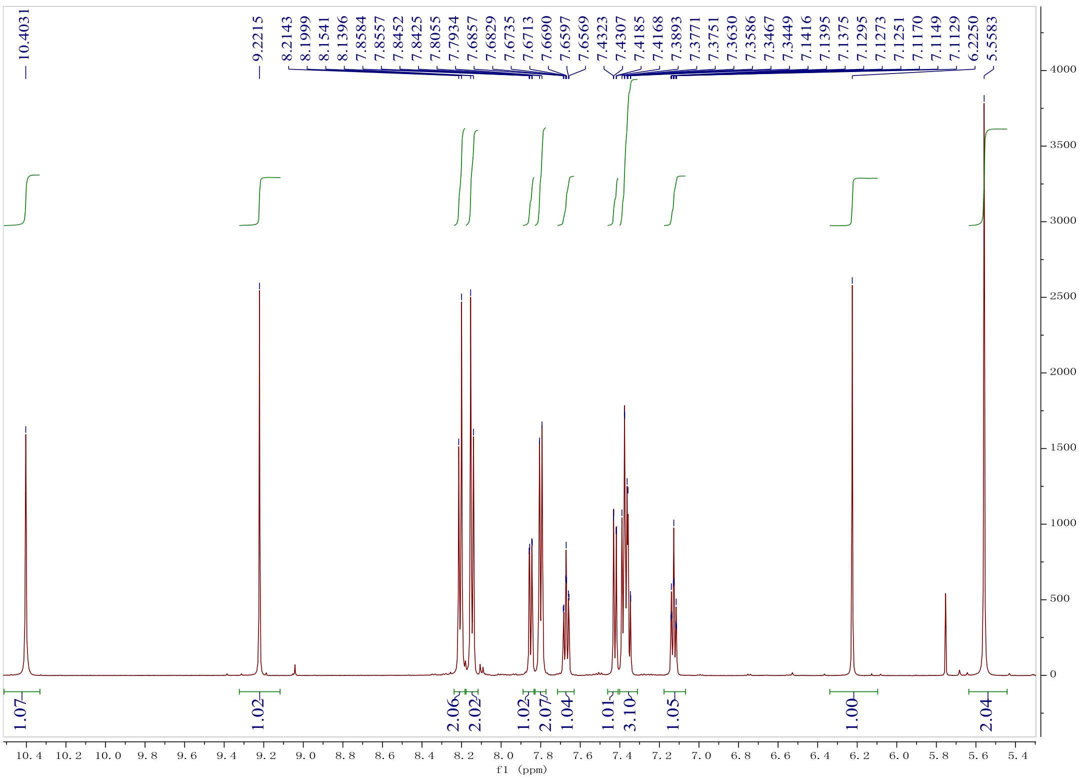

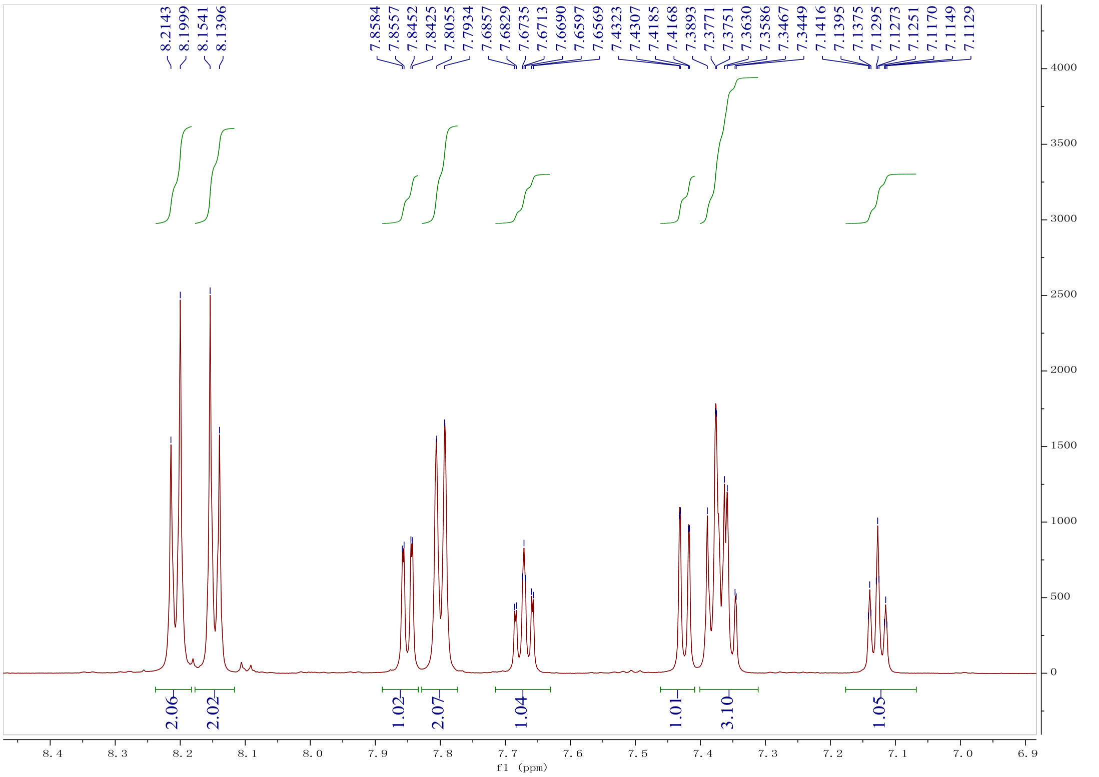
8.2143
8.1999
8.1541
8.1396
7.8584
7.8557
7.8452
7.8425
7.8055
7.7934
7.6857
7.6829
7.6735
7.6713
7.6690
7.6597
7.6569
7.4323
7.4307
7.4185
7.4168
7.3893
7.3771
7.3751
7.3630
7.3586
7.3467
7.3449
7.1416
7.1395
7.1375
7.1295
7.1273
7.1251
7.1170
7.1149
7.1129
2.06
2.02
1.02
2.07
1.04
1.01
3.10
1.05
f1 (ppm)

^{13}C NMR of 5a

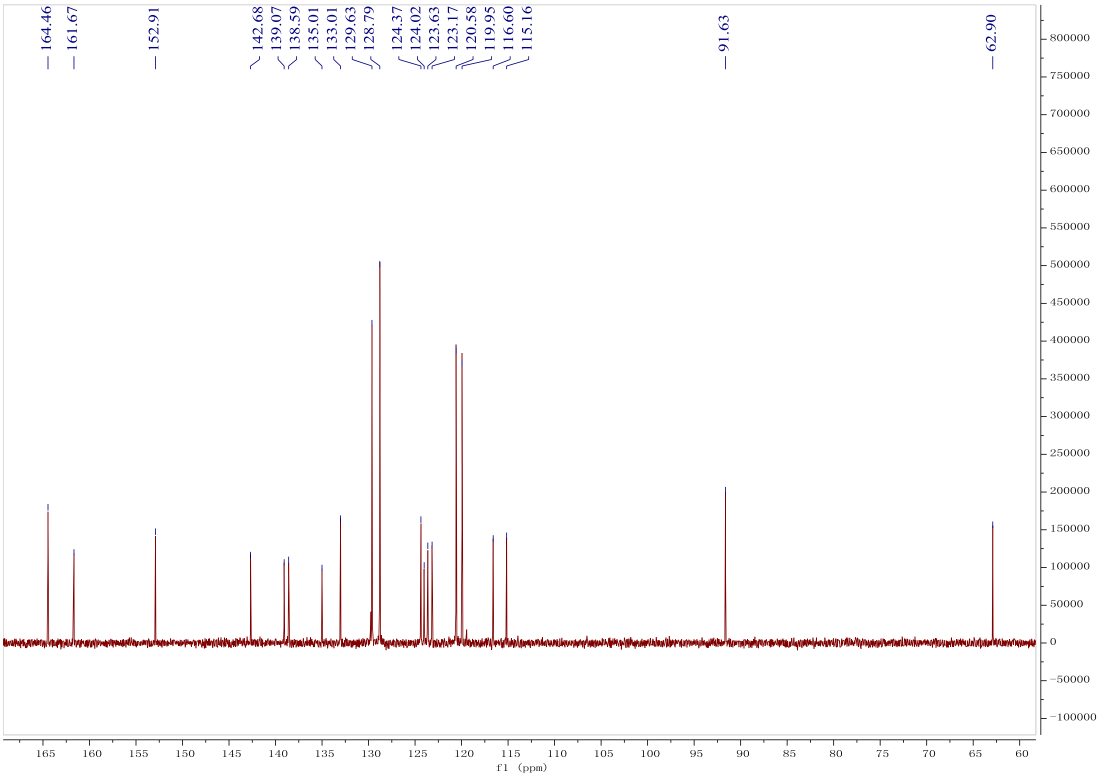
164.46
161.67
152.91
142.68
139.07
138.59
135.01
133.01
129.63
128.79
124.37
124.02
123.63
123.17
120.58
119.95
116.60
115.16
91.63
62.90
f1 (ppm)

5b

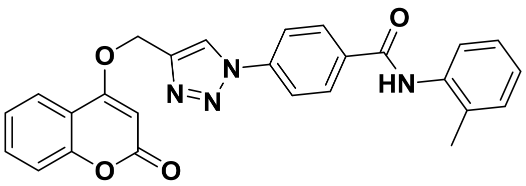

4-(4-(((2-oxo-2*H*-chromen-4-yl)oxy)methyl)-1*H*-1,2,3-triazol-1-yl)-*N*-(*o*-tolyl)benzamide

HRMS of 5b

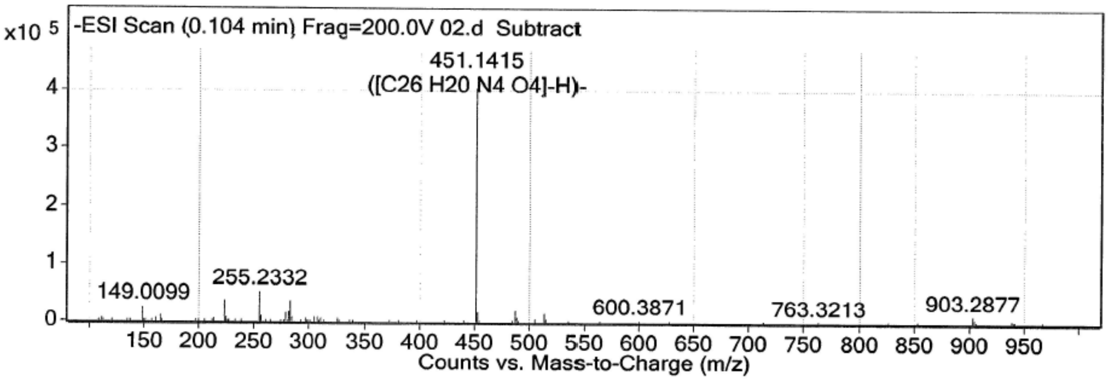

^{1}H NMR of 5b

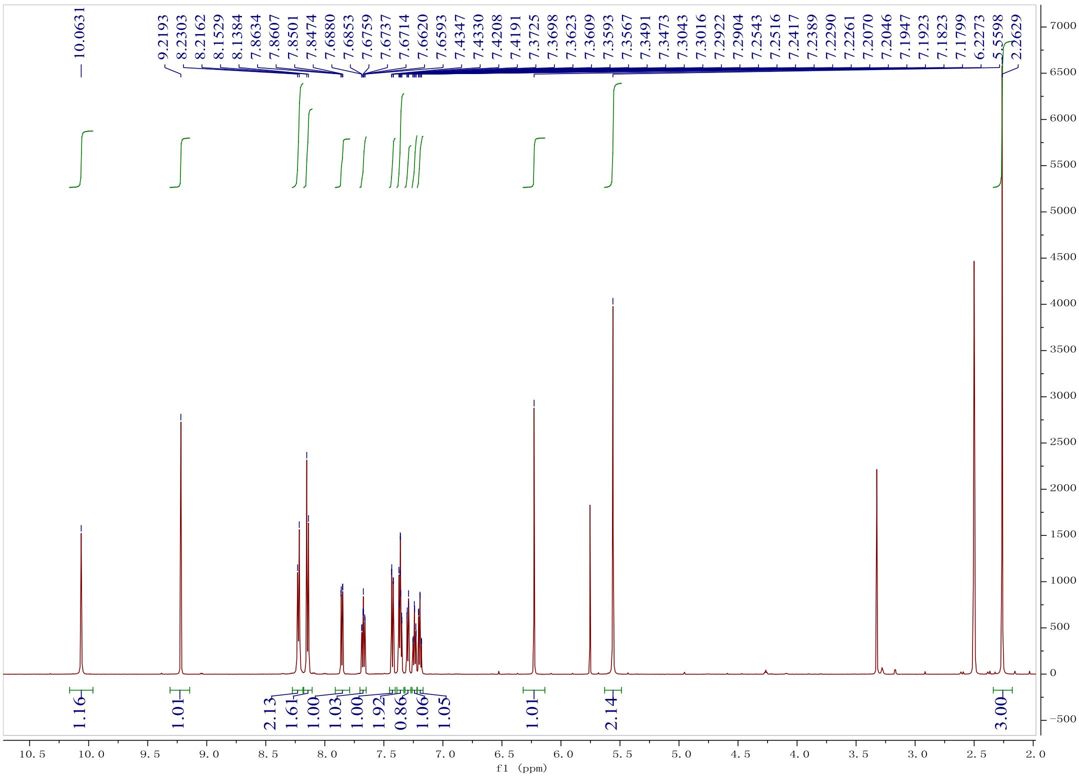

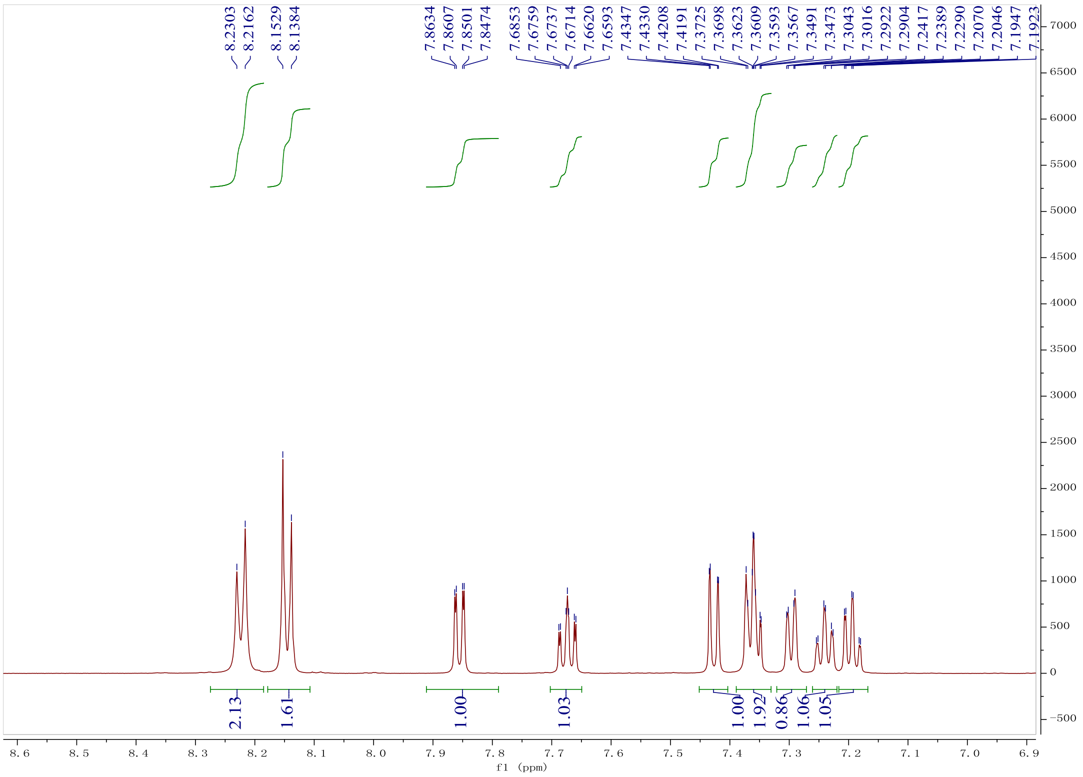
8.2303
8.2162
8.1529
8.1384
7.8634
7.8607
7.8501
7.8474
7.6853
7.6759
7.6737
7.6714
7.6620
7.6593
7.4347
7.4330
7.4208
7.4191
7.3725
7.3698
7.3623
7.3609
7.3593
7.3567
7.3491
7.3473
7.3043
7.3016
7.2922
7.2904
7.2417
7.2389
7.2290
7.2070
7.2046
7.1947
7.1923
2.13
1.61
1.00
1.03
1.00
1.92
0.86
1.06
1.05
f1 (ppm)

^{13}C NMR of 5b

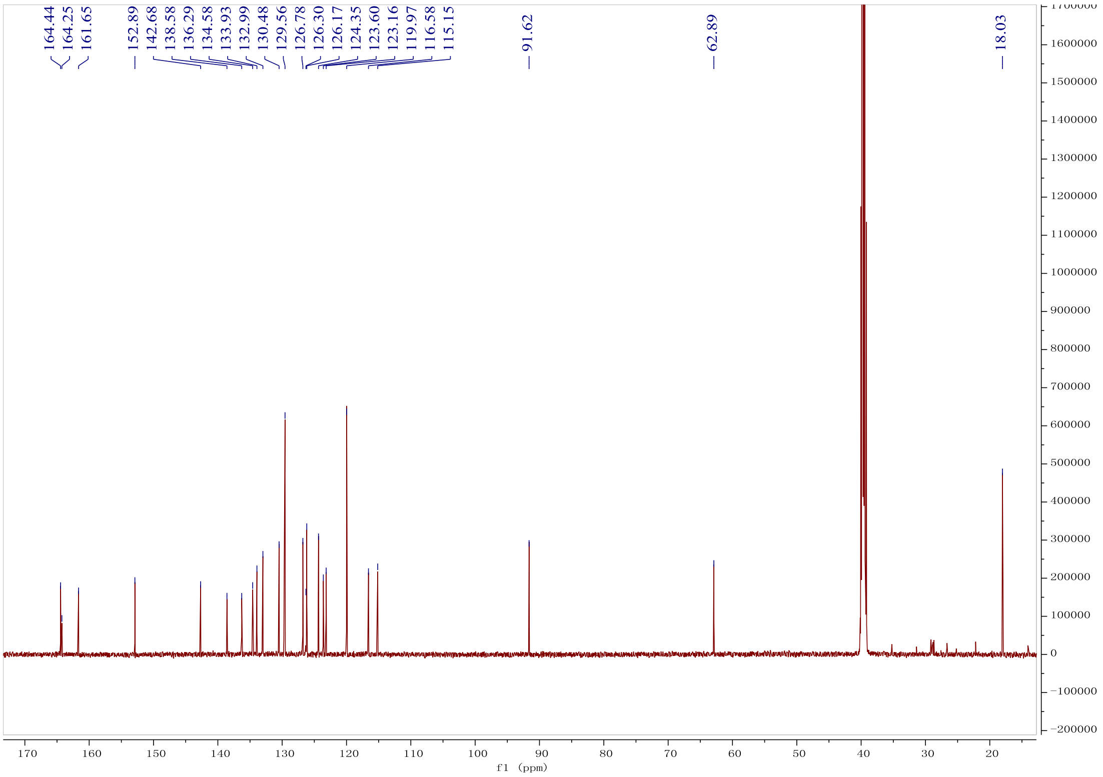
164.44
164.25
161.65
152.89
142.68
138.58
136.29
134.58
133.93
132.99
130.48
129.56
126.78
126.30
126.17
124.35
123.60
123.16
119.97
116.58
115.15
91.62
62.89
18.03
f1 (ppm)

5c

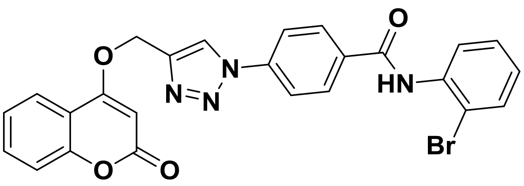

***N*-(2-bromophenyl)-4-(4-(((2-oxo-2*H*-chromen-4-yl)oxy)methyl)-1*H*-1,2,3-triazol-1-yl)benzamide**

HRMS of 5c

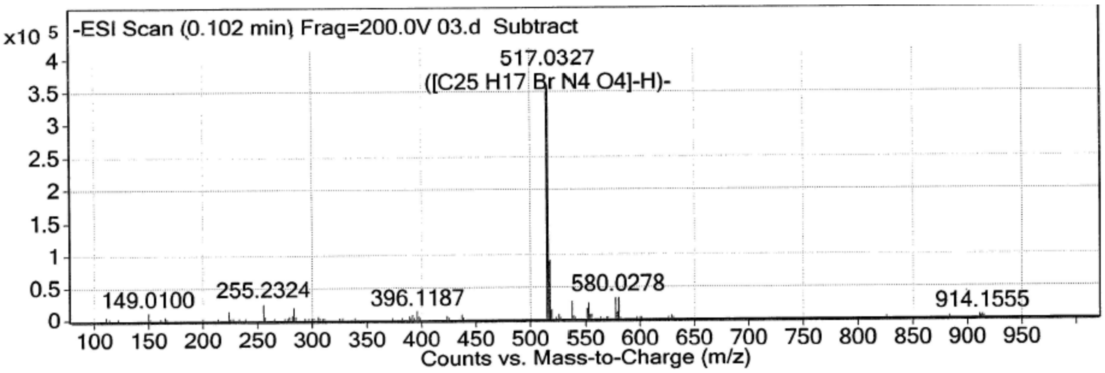

^{1}H NMR of 5c

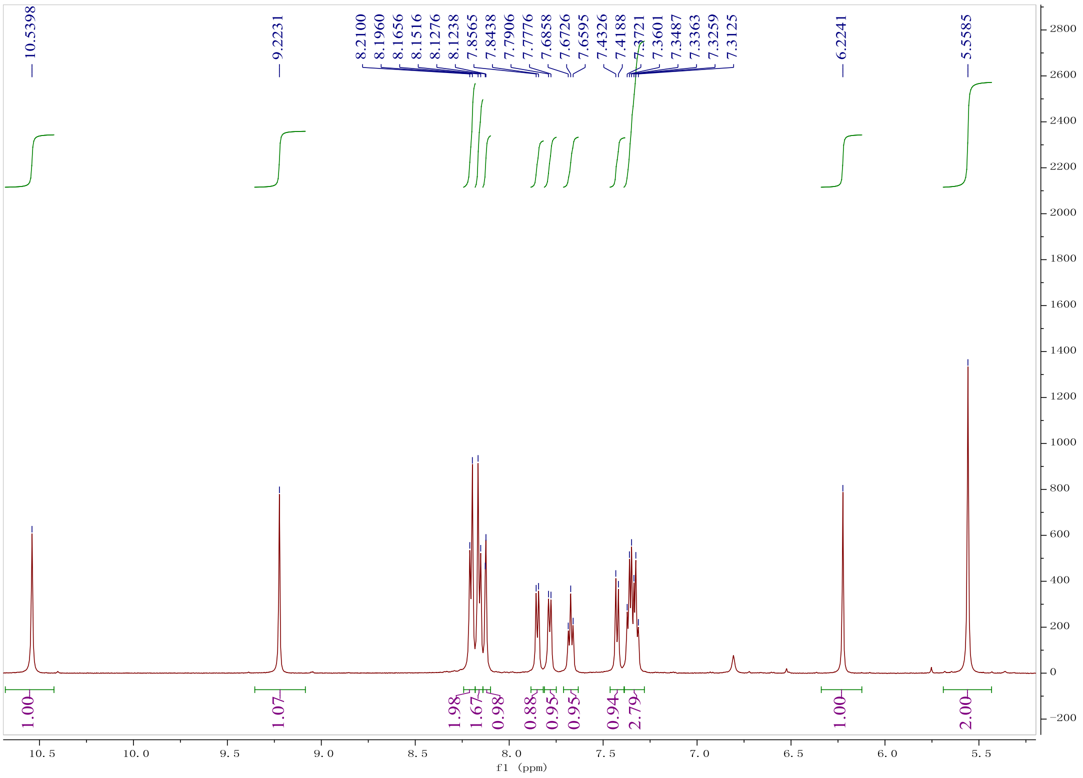

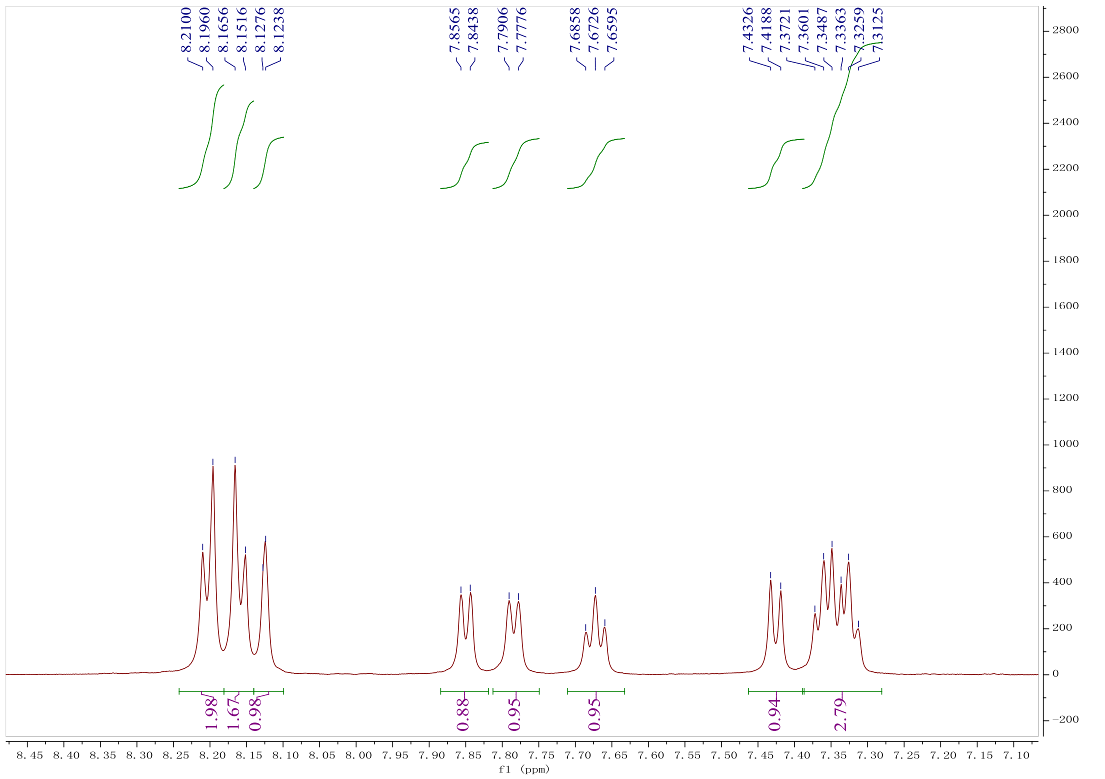
8.2100
8.1960
8.1656
8.1516
8.1276
8.1238
7.8565
7.8438
7.7906
7.7776
7.6858
7.6726
7.6595
7.4326
7.4188
7.3721
7.3601
7.3487
7.3363
7.3259
7.3125
1.98
1.67
0.98
0.88
0.95
0.95
0.94
2.79
f1 (ppm)

^{13}C NMR of 5c

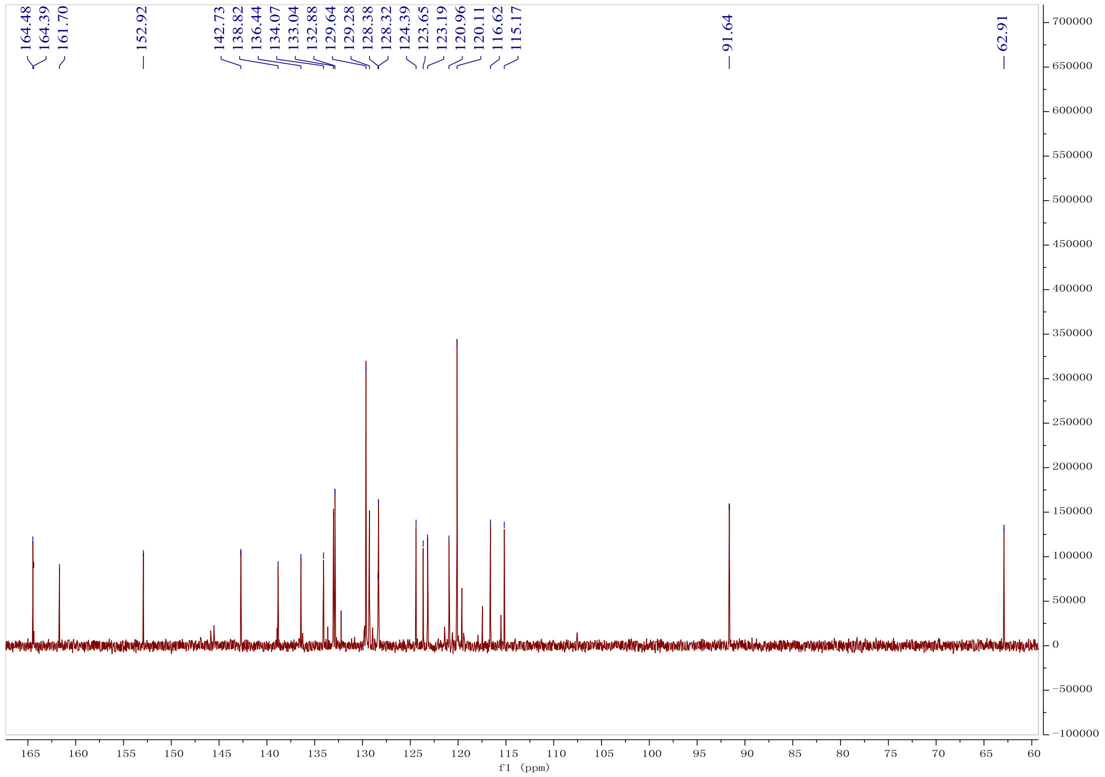
164.48
164.39
161.70
152.92
142.73
138.82
136.44
134.07
133.04
132.88
129.64
129.28
128.38
128.32
124.39
123.65
123.19
120.96
120.11
116.62
115.17
91.64
62.91
f1 (ppm)

5d

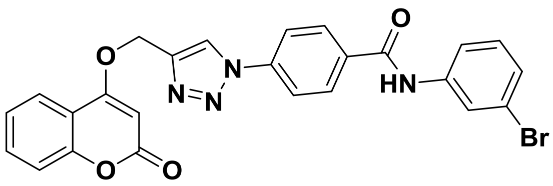

***N*-(3-bromophenyl)-4-(4-(((2-oxo-2*H*-chromen-4-yl)oxy)methyl)-1*H*-1,2,3-triazol-1-yl)benzamide**

HRMS of 5d

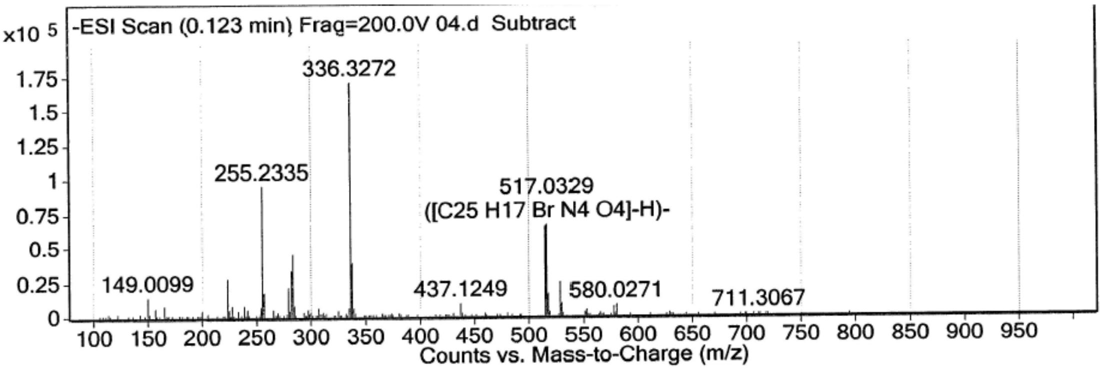

^{1}H NMR of 5d

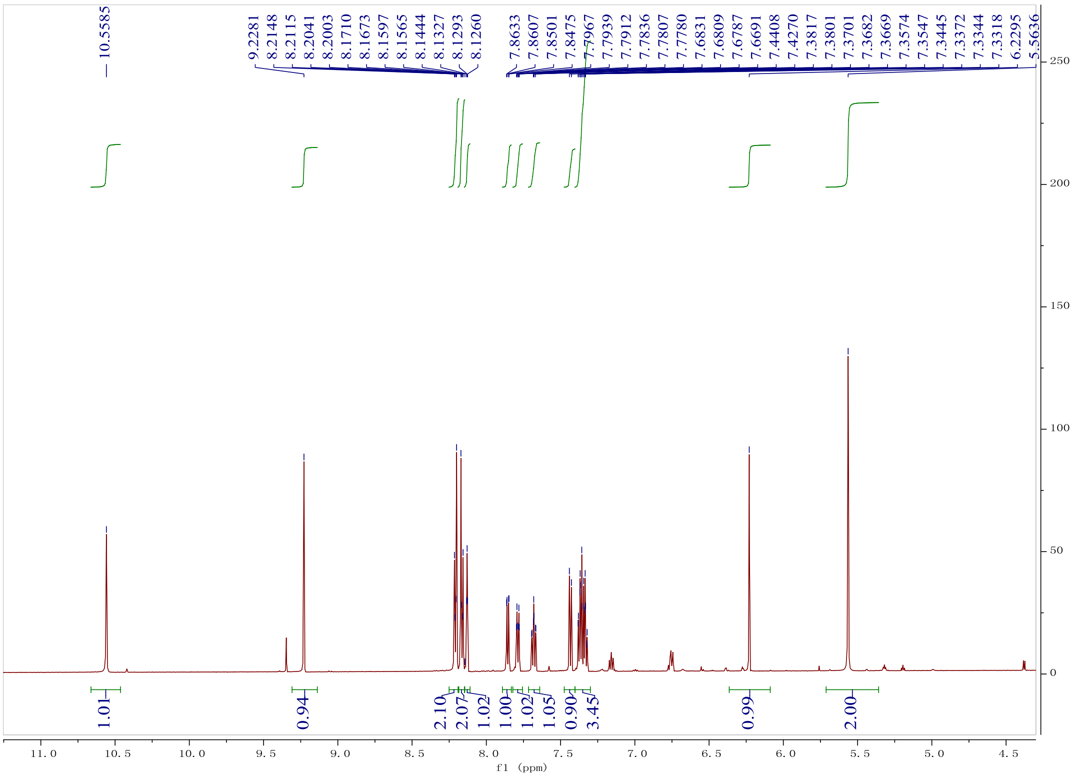

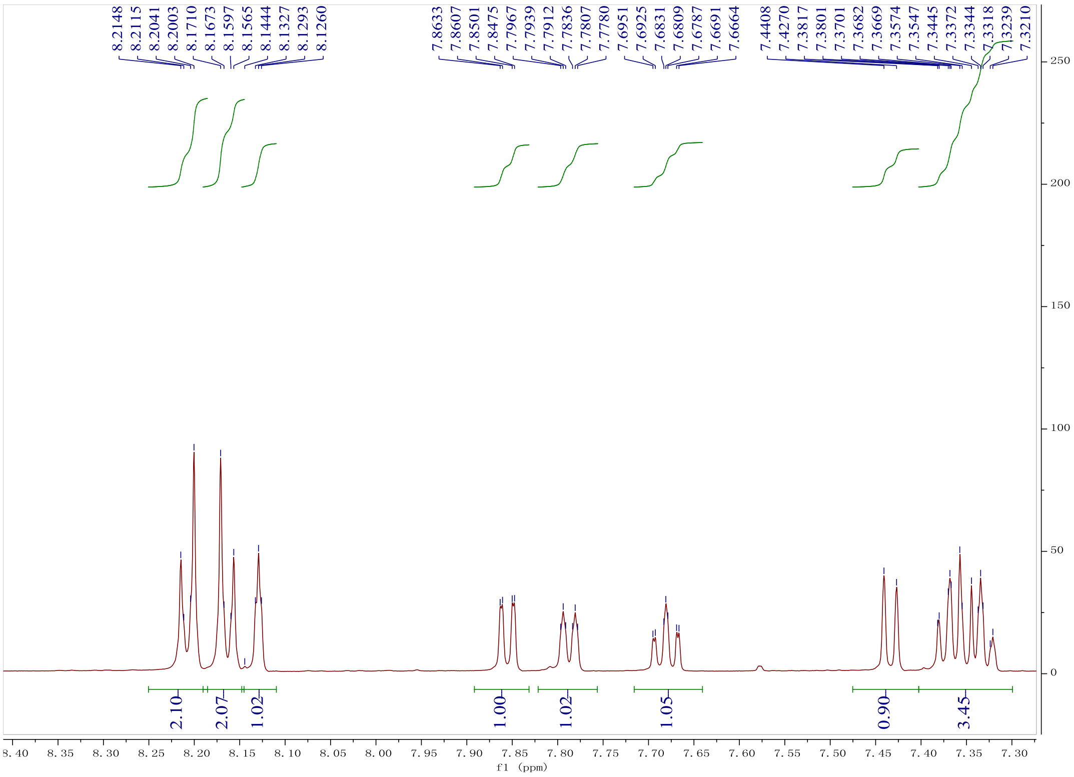
8.2148
8.2115
8.2041
8.2003
8.1710
8.1673
8.1597
8.1565
8.1444
8.1327
8.1293
8.1260
7.8633
7.8607
7.8501
7.8475
7.7967
7.7939
7.7912
7.7836
7.7807
7.7780
7.6951
7.6925
7.6831
7.6809
7.6787
7.6691
7.6664
7.4408
7.4270
7.3817
7.3801
7.3701
7.3682
7.3669
7.3574
7.3547
7.3445
7.3372
7.3344
7.3318
7.3239
7.3210
2.10
2.07
1.02
1.00
1.02
1.05
0.90
3.45
f1 (ppm)

^{13}C NMR of 5d

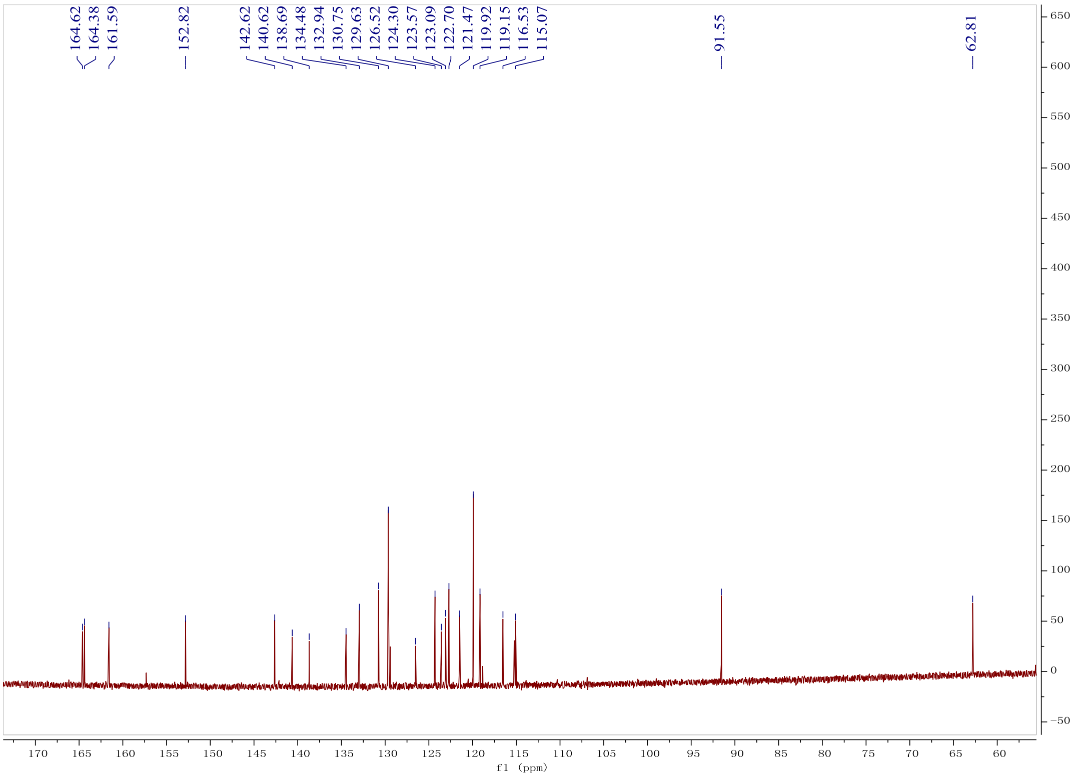
164.62
164.38
161.59
152.82
142.62
140.62
138.69
134.48
132.94
130.75
129.63
126.52
124.30
123.57
123.09
122.70
121.47
119.92
119.15
116.53
115.07
91.55
62.81
f1 (ppm)

5e

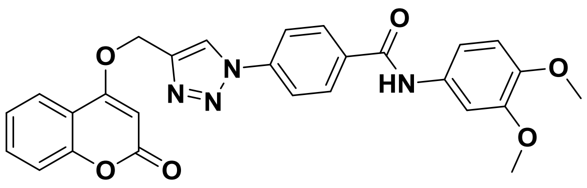

***N*-(3,4-dimethoxyphenyl)-4-(4-(((2-oxo-2*H*-chromen-4-yl)oxy)methyl)-1*H*-1,2,3-triazol-1-yl)benzamide**

HRMS of 5e

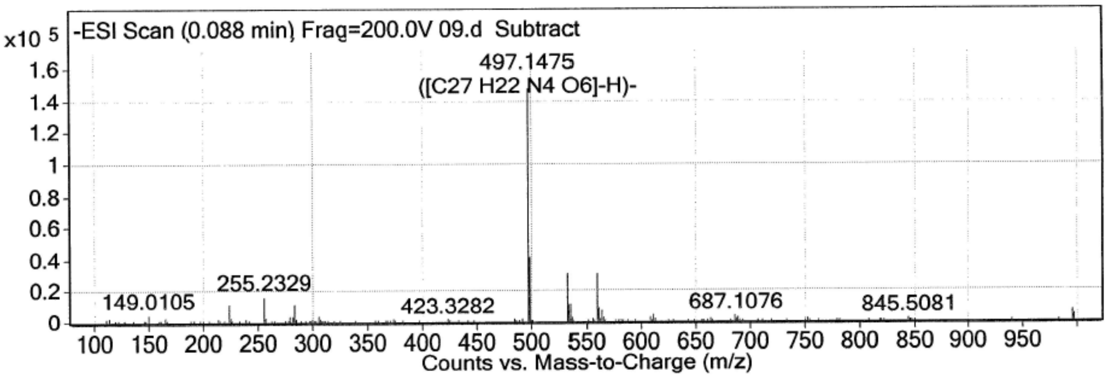

^{1}H NMR of 5e

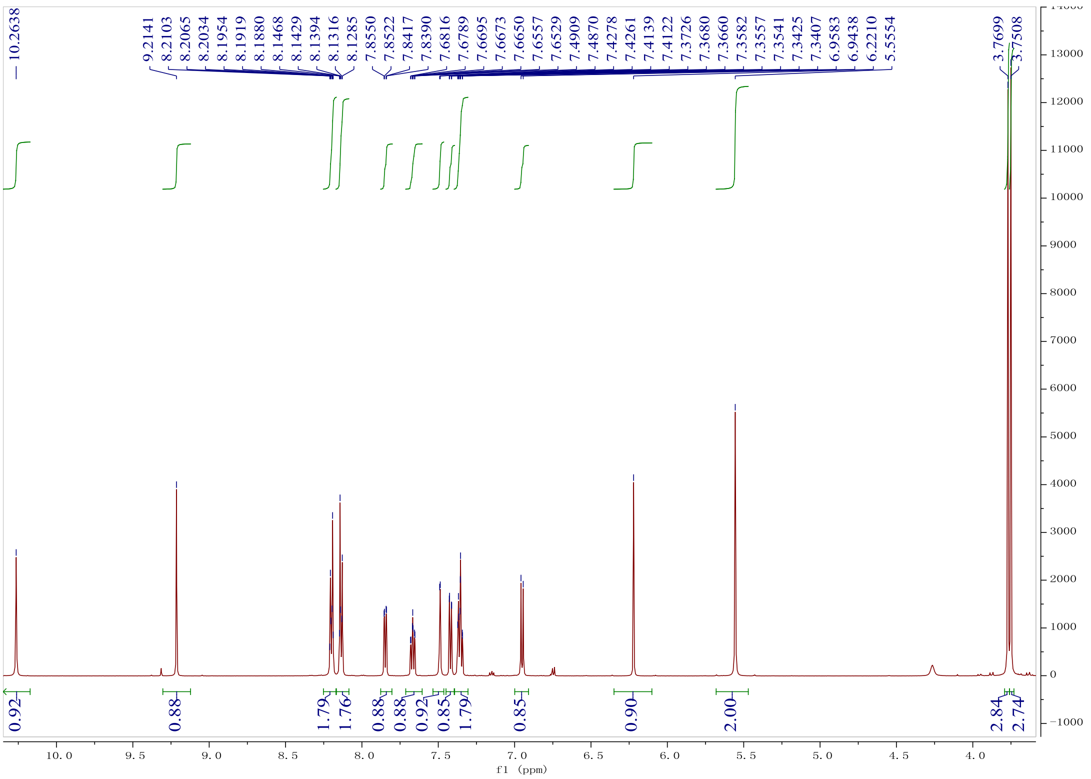

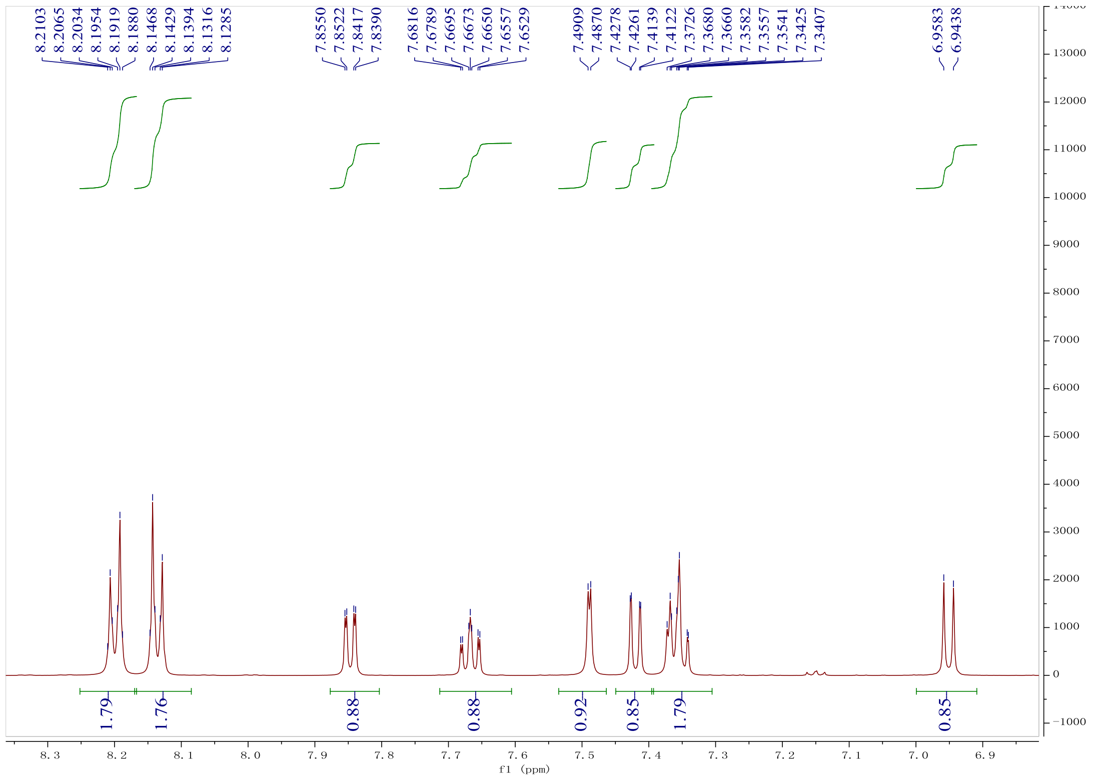

8.2103
8.2065
8.2034
8.1954
8.1919
8.1880
8.1468
8.1429
8.1394
8.1316
8.1285
7.8550
7.8522
7.8417
7.8390
7.6816
7.6789
7.6695
7.6673
7.6650
7.6557
7.6529
7.4909
7.4870
7.4278
7.4261
7.4139
7.4122
7.3726
7.3680
7.3660
7.3582
7.3557
7.3541
7.3425
7.3407
6.9583
6.9438
1.79
1.76
0.88
0.88
0.92
0.85
1.79
0.85
f1 (ppm)

^{13}C NMR of 5e

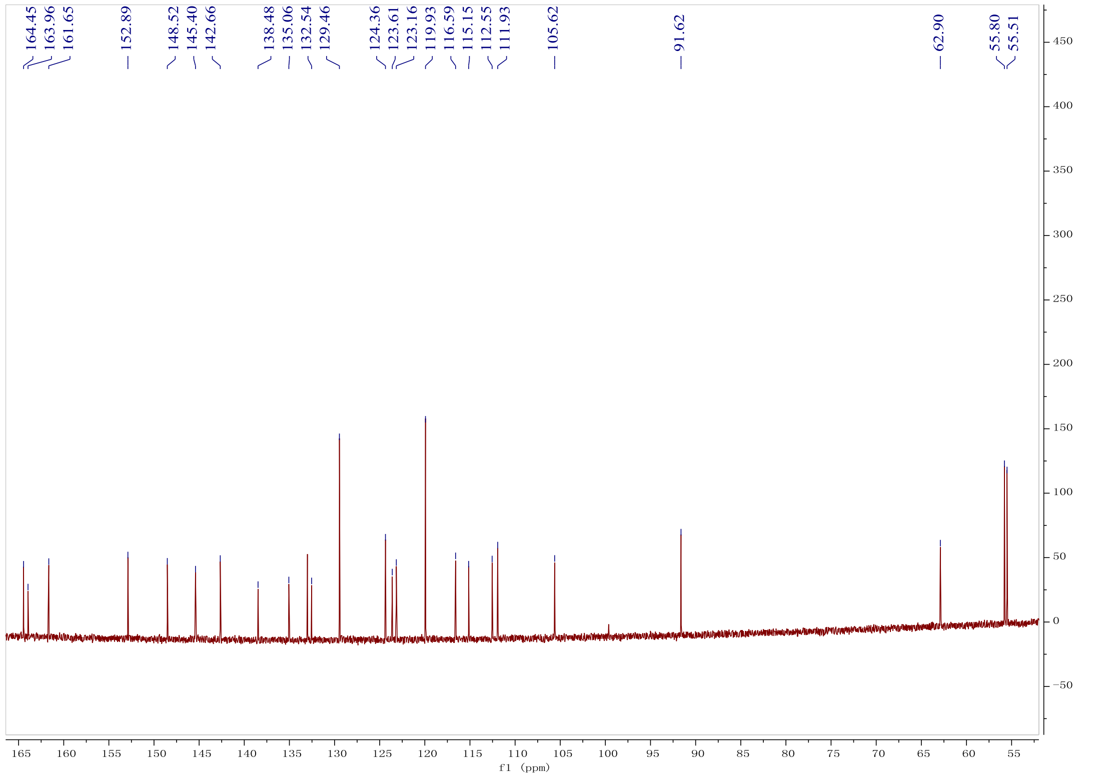

164.45
163.96
161.65
152.89
148.52
145.40
142.66
138.48
135.06
132.54
129.46
124.36
123.61
123.16
119.93
116.59
115.15
112.55
111.93
105.62
91.62
62.90
55.80
55.51
f1 (ppm)

5f

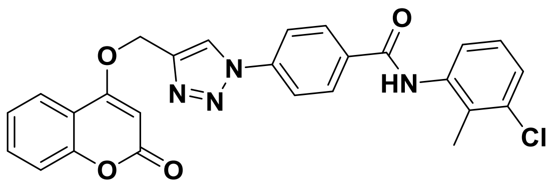

***N*-(3-chloro-2-methylphenyl)-4-(4-(((2-oxo-2*H*-chromen-4-yl)oxy)methyl)-1*H*-1,2,3-triazol-1-yl)benzamide**

HRMS of 5f

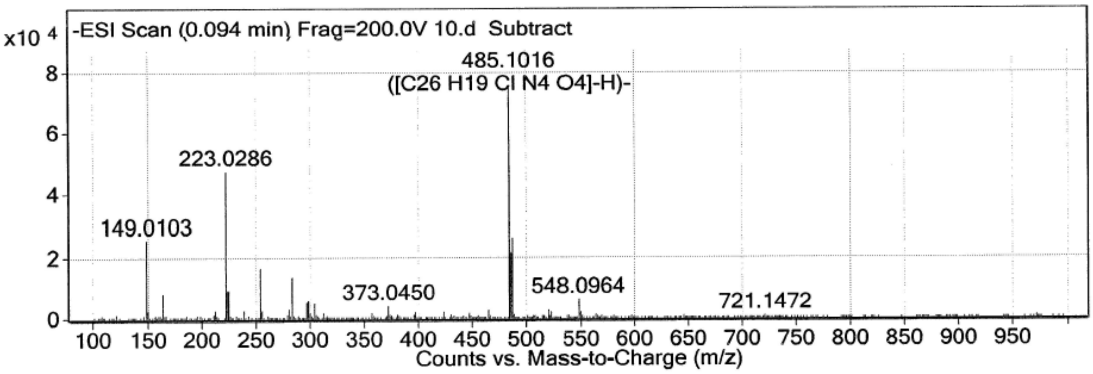

^{1}H NMR of 5f

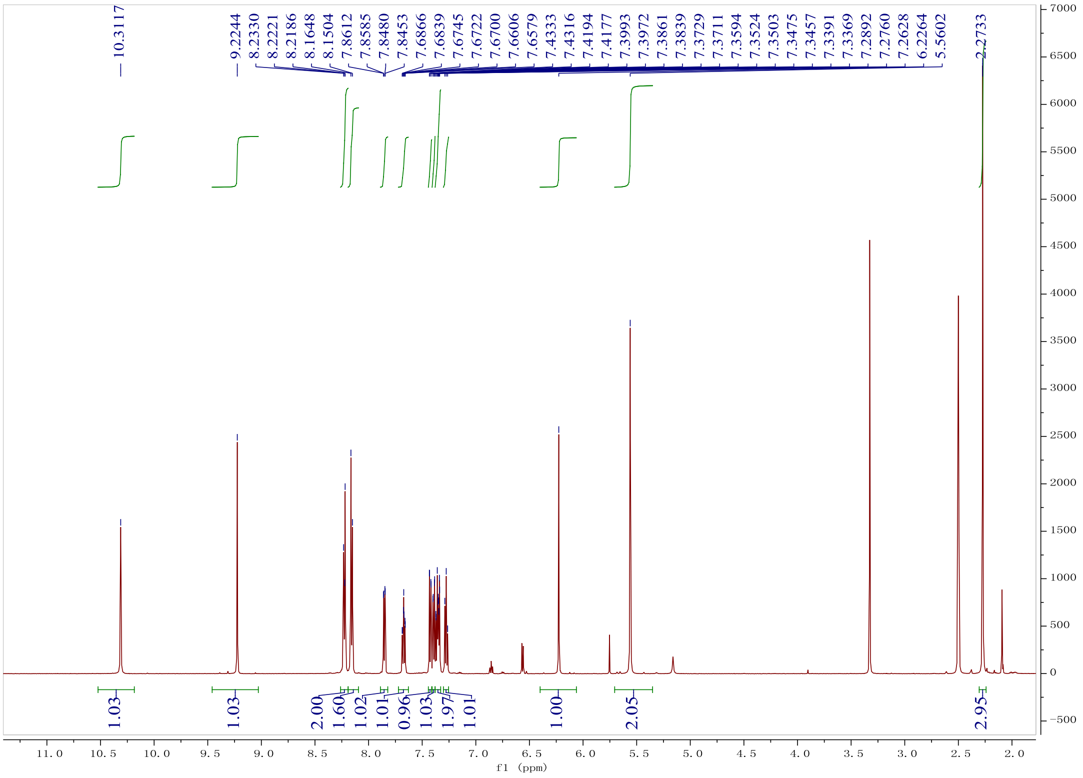

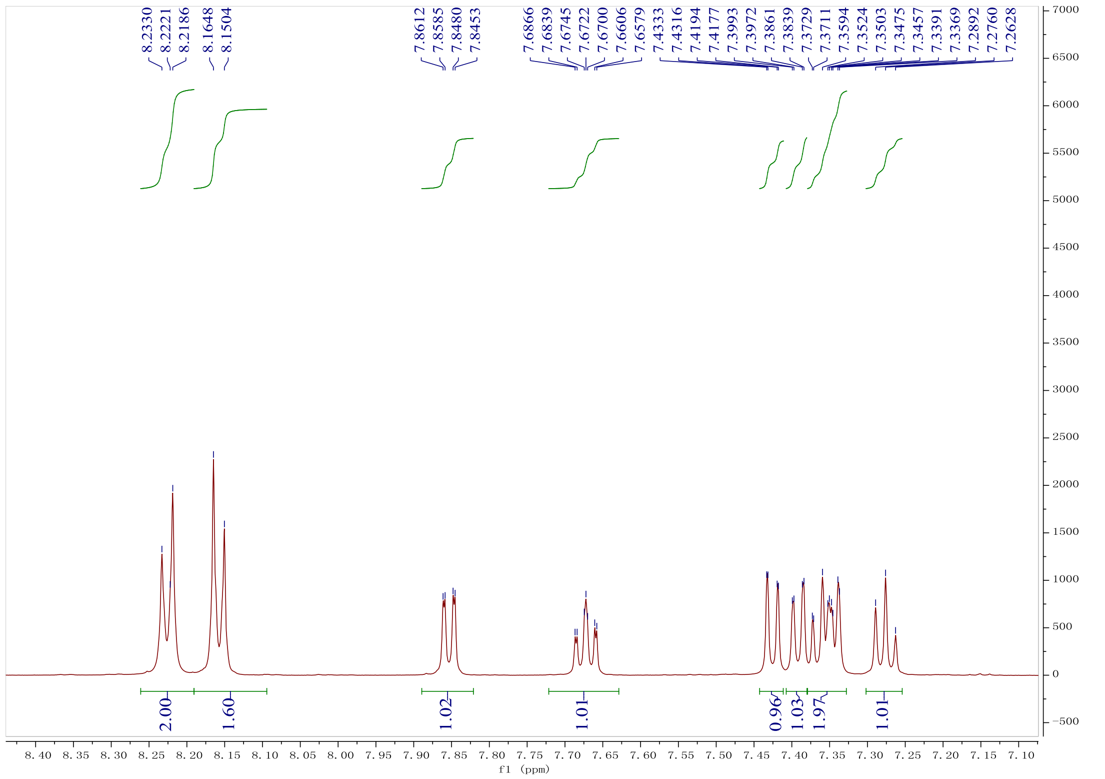
8.2330
8.2221
8.2186
8.1648
8.1504
7.8612
7.8585
7.8480
7.8453
7.6866
7.6839
7.6745
7.6722
7.6700
7.6606
7.6579
7.4333
7.4316
7.4194
7.4177
7.3993
7.3972
7.3861
7.3839
7.3729
7.3711
7.3594
7.3524
7.3503
7.3475
7.3457
7.3391
7.3369
7.2892
7.2760
7.2628
2.00
1.60
1.02
1.01
0.96
1.03
1.97
1.01
f1 (ppm)

^{13}C NMR of 5f

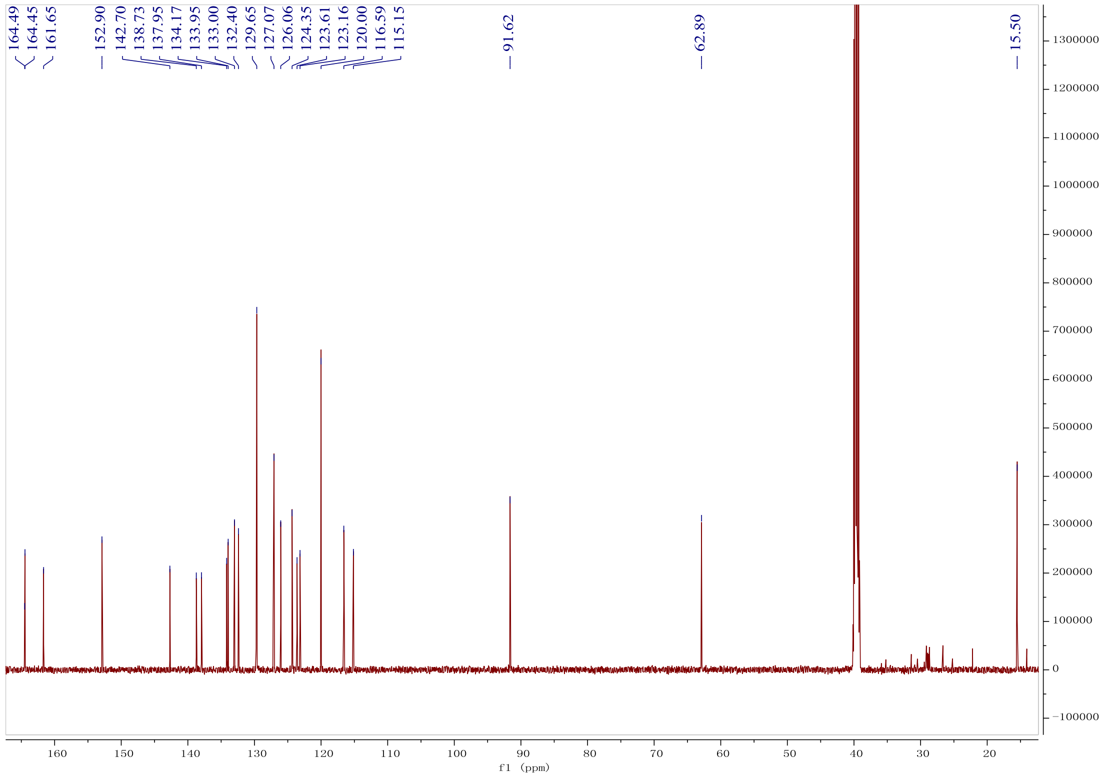
164.49
164.45
161.65
152.90
142.70
138.73
137.95
134.17
133.95
133.00
132.40
129.65
127.07
126.06
124.35
123.61
123.16
120.00
116.59
115.15
91.62
62.89
15.50
f1 (ppm)

5g

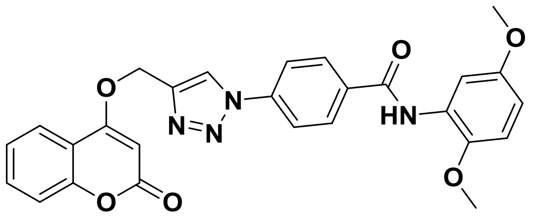

***N*-(2,5-dimethoxyphenyl)-4-(4-(((2-oxo-2*H*-chromen-4-yl)oxy)methyl)-1*H*-1,2,3-triazol-1-yl)benzamide**

HRMS of 5g

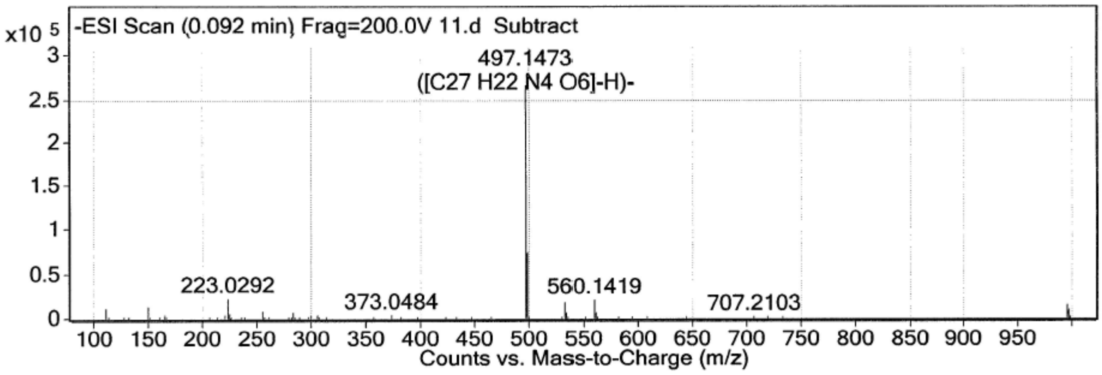

^{1}H NMR of 5g

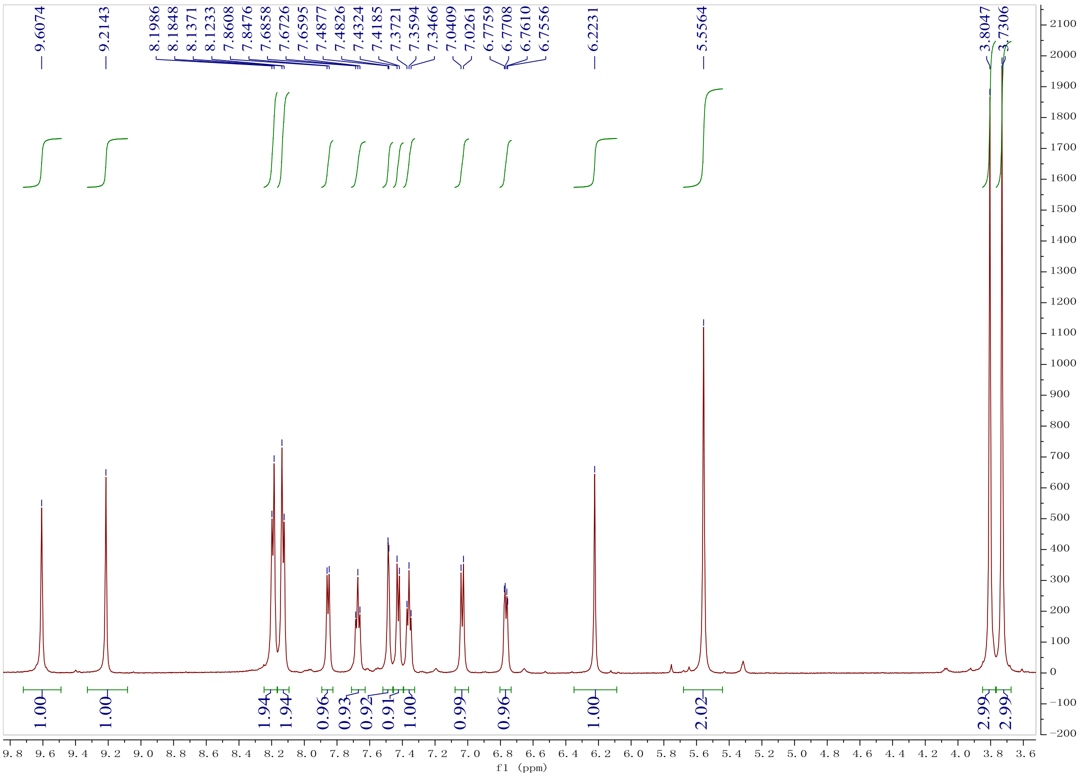

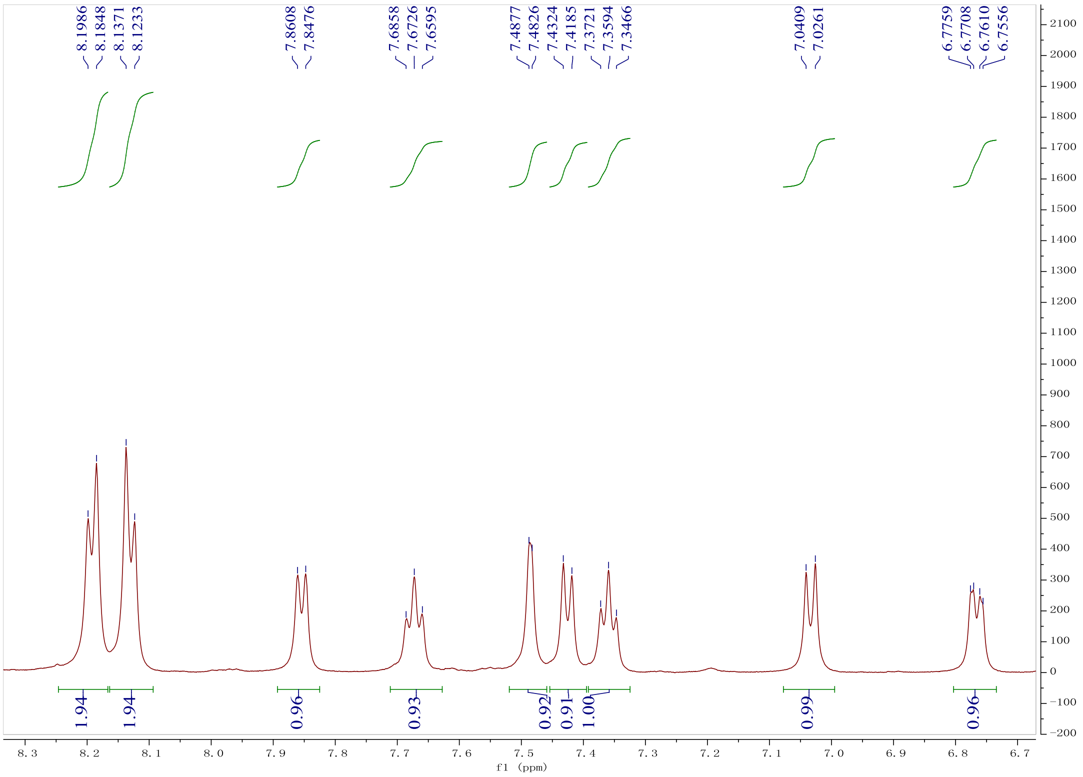

8.1986
8.1848
8.1371
8.1233
7.8608
7.8476
7.6858
7.6726
7.6595
7.4877
7.4826
7.4324
7.4185
7.3721
7.3594
7.3466
7.0409
7.0261
6.7759
6.7708
6.7610
6.7556
1.94
1.94
0.96
0.93
0.92
0.91
1.00
0.99
0.96
f1 (ppm)

^{13}C NMR of 5g

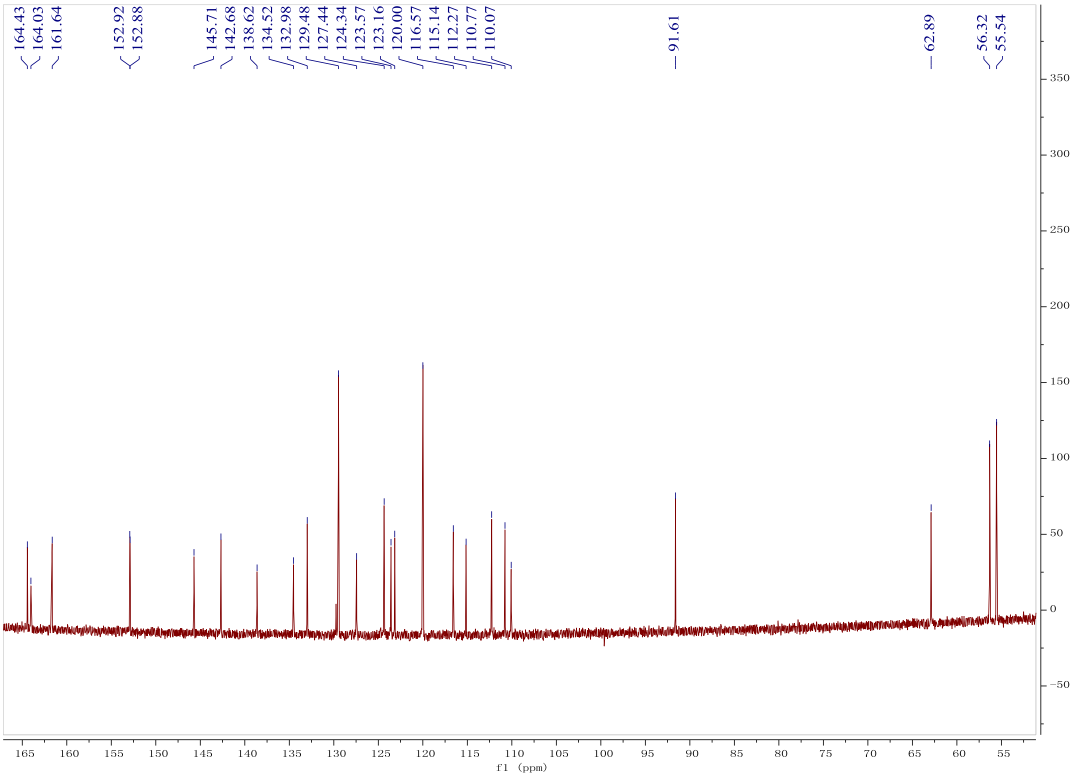

164.43
164.03
161.64
152.92
152.88
145.71
142.68
138.62
134.52
132.98
129.48
127.44
124.34
123.57
123.16
120.00
116.57
115.14
112.27
110.77
110.07
91.61
62.89
56.32
55.54
f1 (ppm)

5h

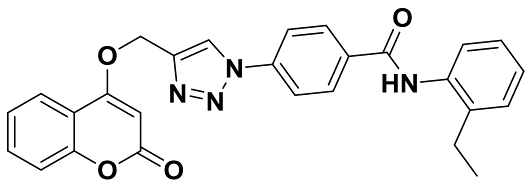

***N*-(2-ethylphenyl)-4-(4-(((2-oxo-2*H*-chromen-4-yl)oxy)methyl)-1*H*-1,2,3-triazol-1-yl)benzamide**

HRMS of 5h

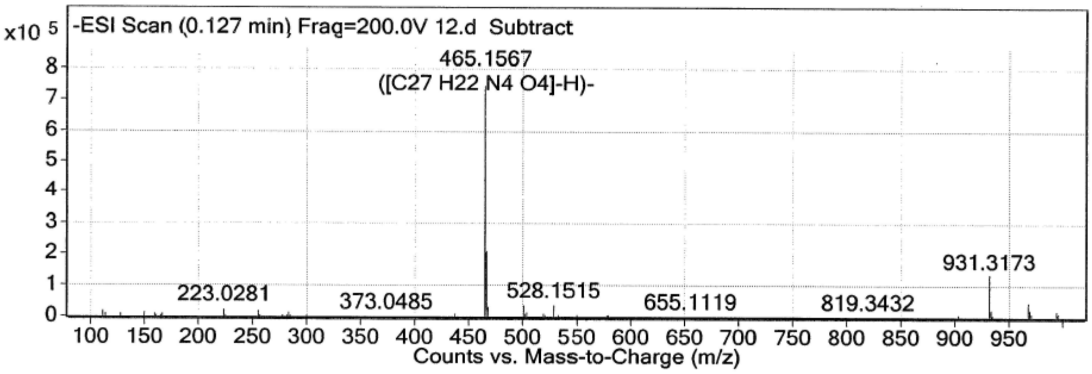

^{1}H NMR of 5h

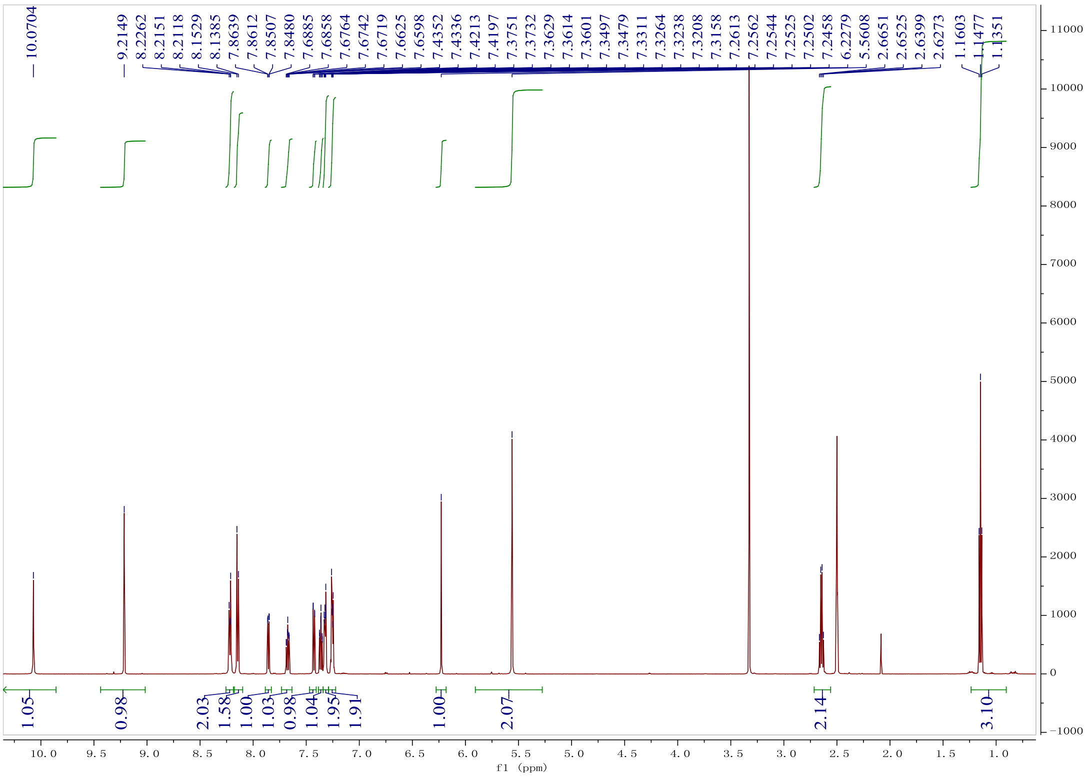

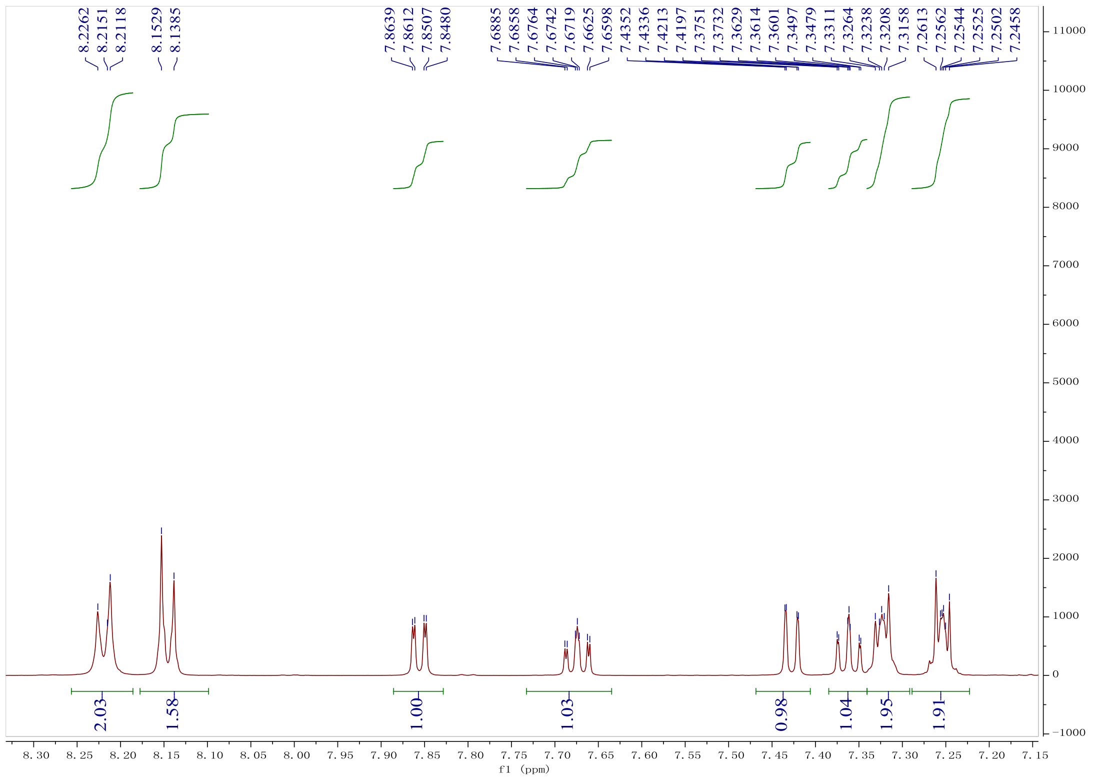

8.2262
8.2151
8.2118
8.1529
8.1385
7.8639
7.8612
7.8507
7.8480
7.6885
7.6858
7.6764
7.6742
7.6719
7.6625
7.6598
7.4352
7.4336
7.4213
7.4197
7.3751
7.3732
7.3629
7.3614
7.3601
7.3497
7.3479
7.3311
7.3264
7.3238
7.3208
7.3158
7.2613
7.2562
7.2544
7.2525
7.2502
7.2458
2.03
1.58
1.00
1.03
0.98
1.04
1.95
1.91
f1 (ppm)

^{13}C NMR of 5h

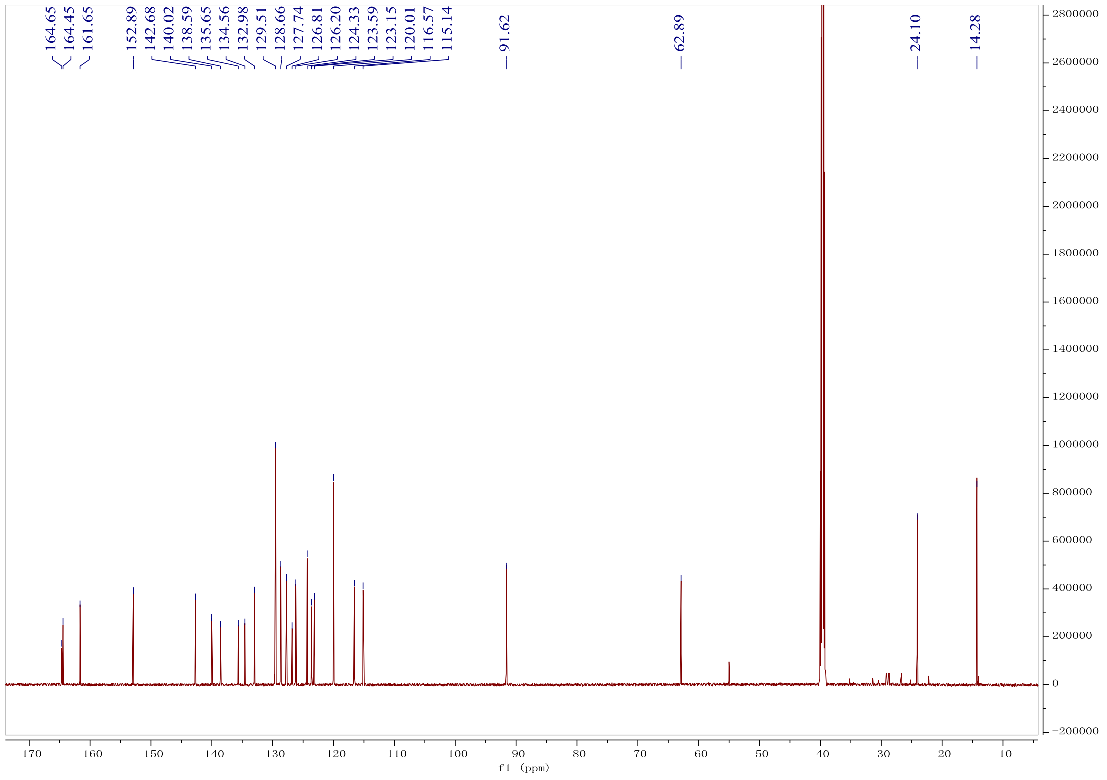

164.65
164.45
161.65
152.89
142.68
140.02
138.59
135.65
134.56
132.98
129.51
128.66
127.74
126.81
126.20
124.33
123.59
123.15
120.01
116.57
115.14
91.62
62.89
24.10
14.28
f1 (ppm)

5i

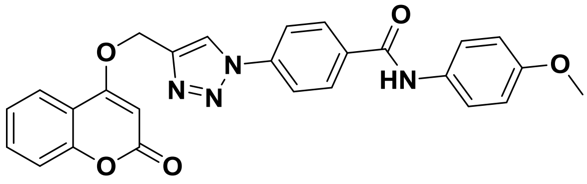

***N*-(4-methoxyphenyl)-4-(4-(((2-oxo-2*H*-chromen-4-yl)oxy)methyl)-1*H*-1,2,3-triazol-1-yl)benzamide**

HRMS of 5i

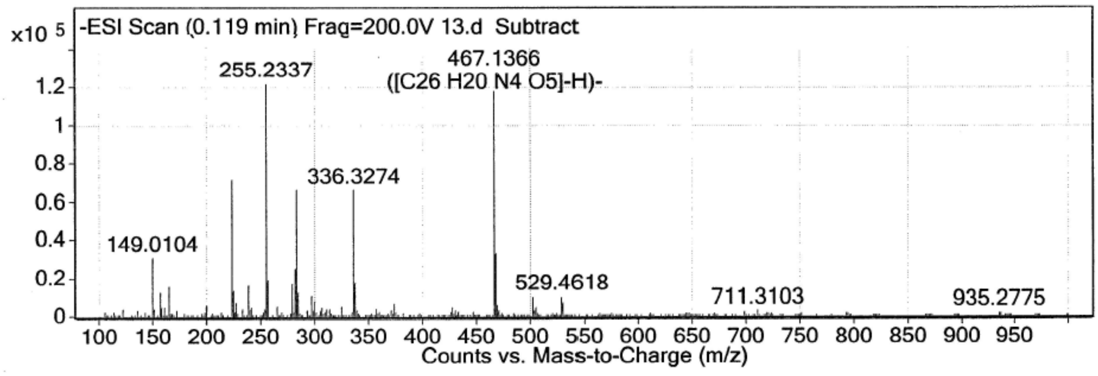

^{1}H NMR of 5i

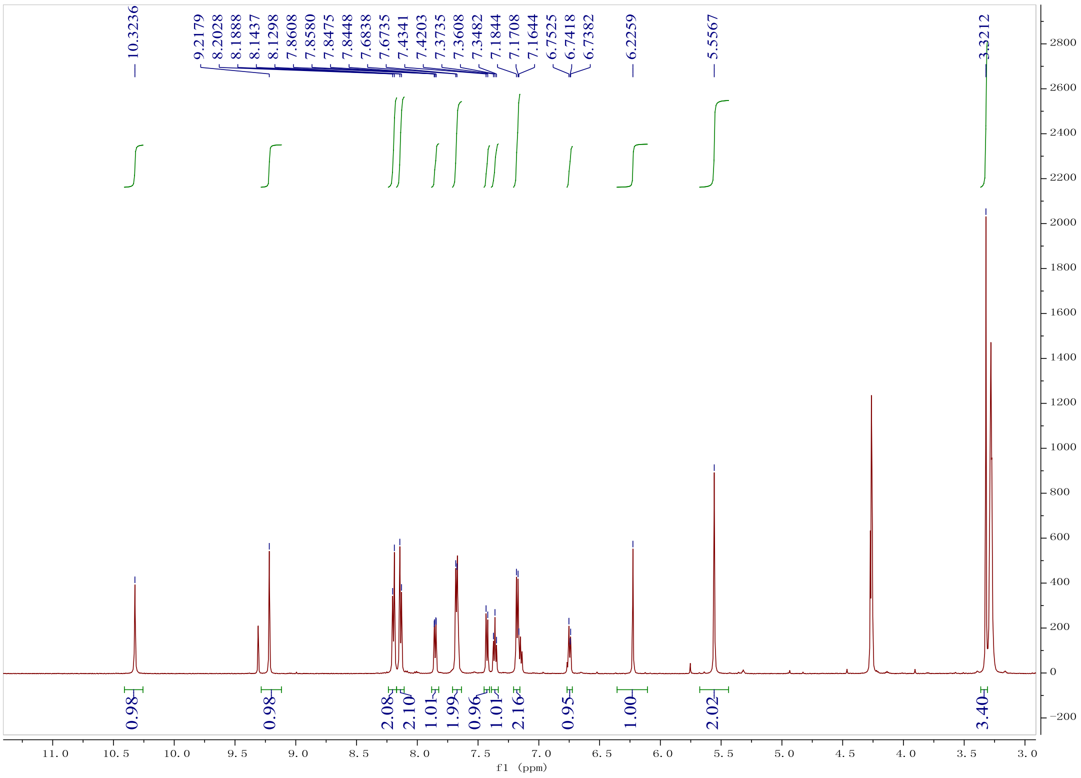

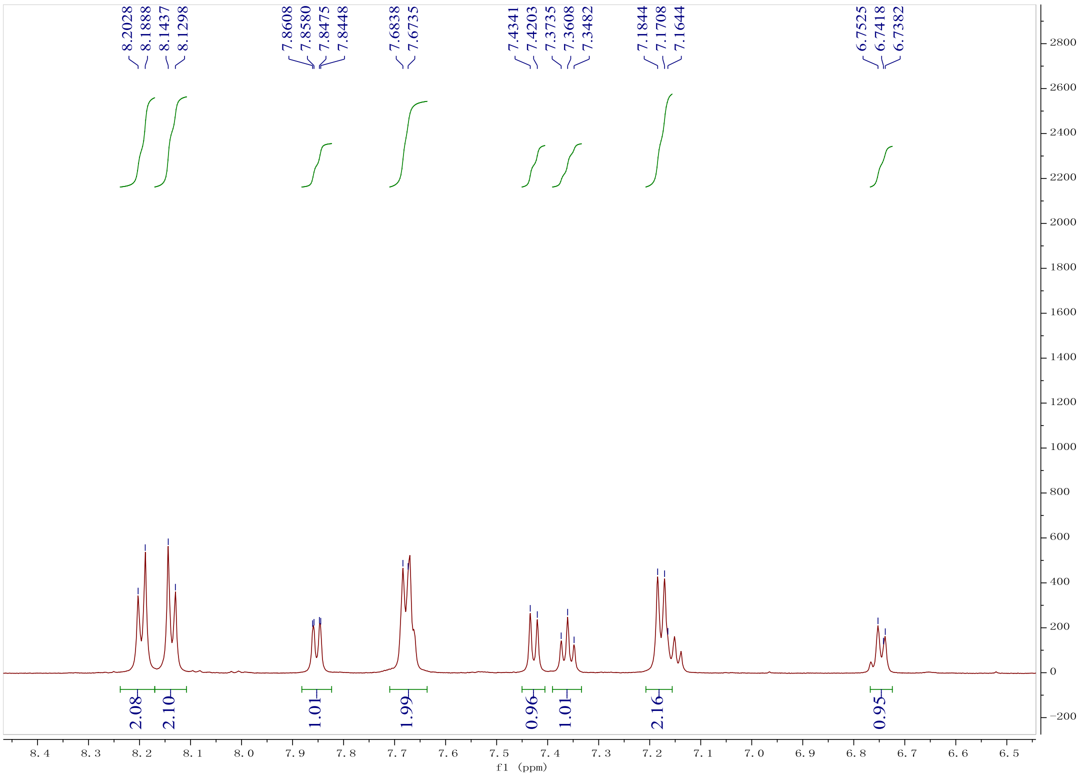
8.2028
8.1888
8.1437
8.1298
7.8608
7.8580
7.8475
7.8448
7.6838
7.6735
7.4341
7.4203
7.3735
7.3608
7.3482
7.1844
7.1708
7.1644
6.7525
6.7418
6.7382
2.08
2.10
1.01
1.99
0.96
1.01
2.16
0.95
f1 (ppm)

^{13}C NMR of 5i

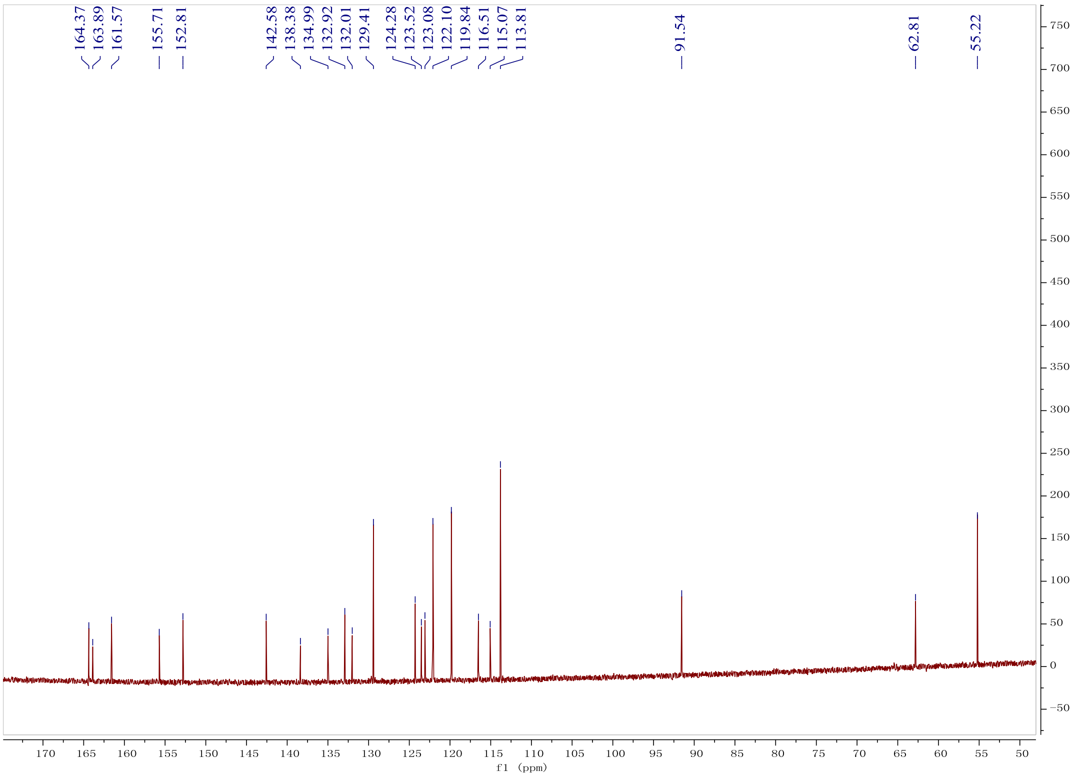
164.37
163.89
161.57
155.71
152.81
142.58
138.38
134.99
132.92
132.01
129.41
124.28
123.52
123.08
122.10
119.84
116.51
115.07
113.81
91.54
62.81
55.22
f1 (ppm)

5j

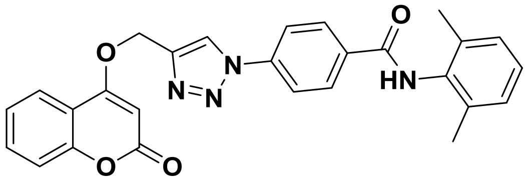

N-(2,6-dimethylphenyl)-4-(4-(((2-oxo-2H-chromen-4-yl)oxy)methyl)-1H-1,2,3-triazol-1-yl)benzamide

HRMS of 5j

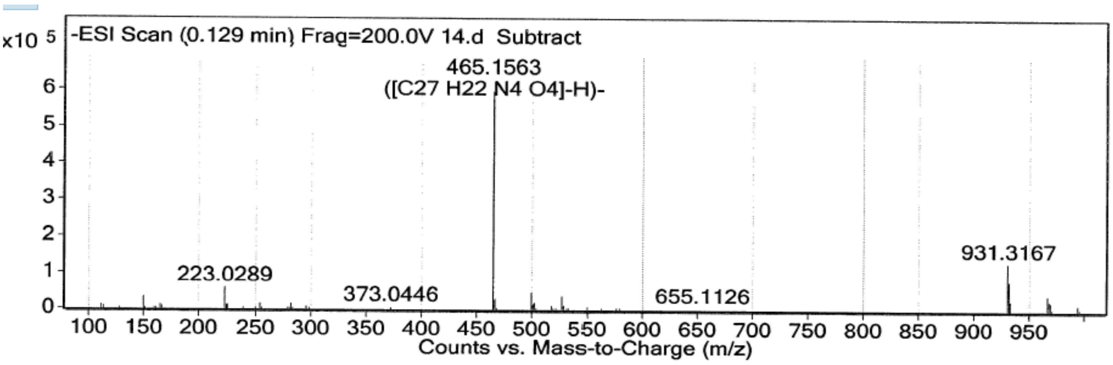

^{1}H NMR of 5j

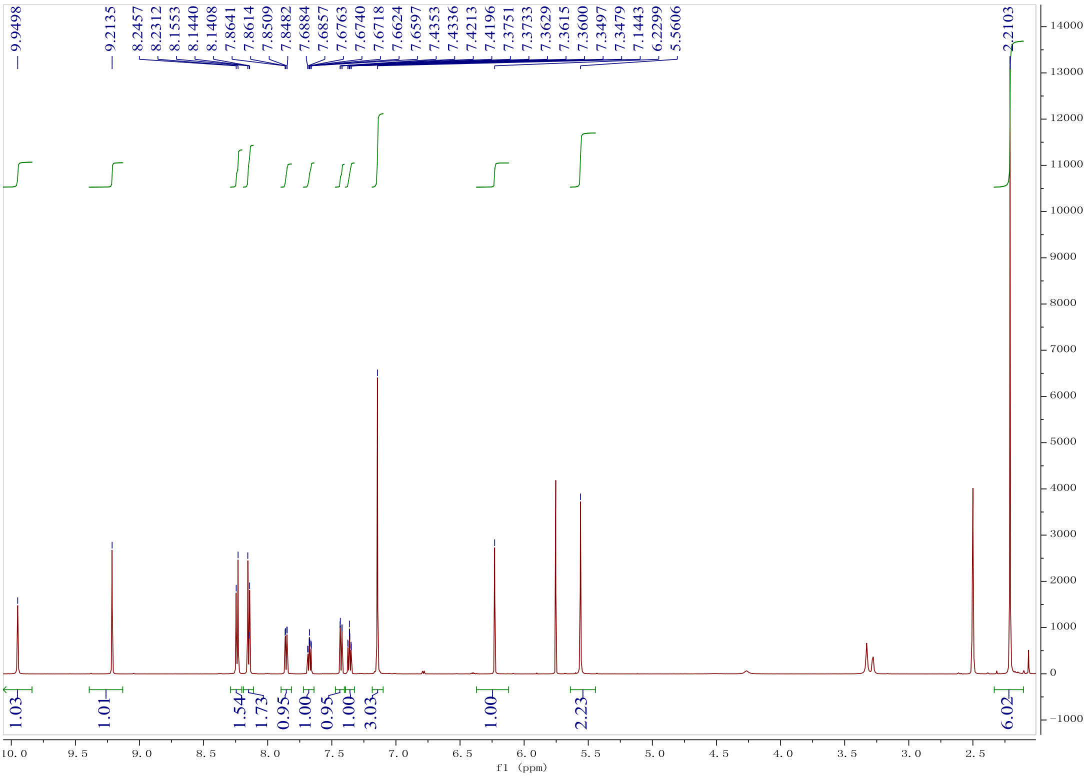

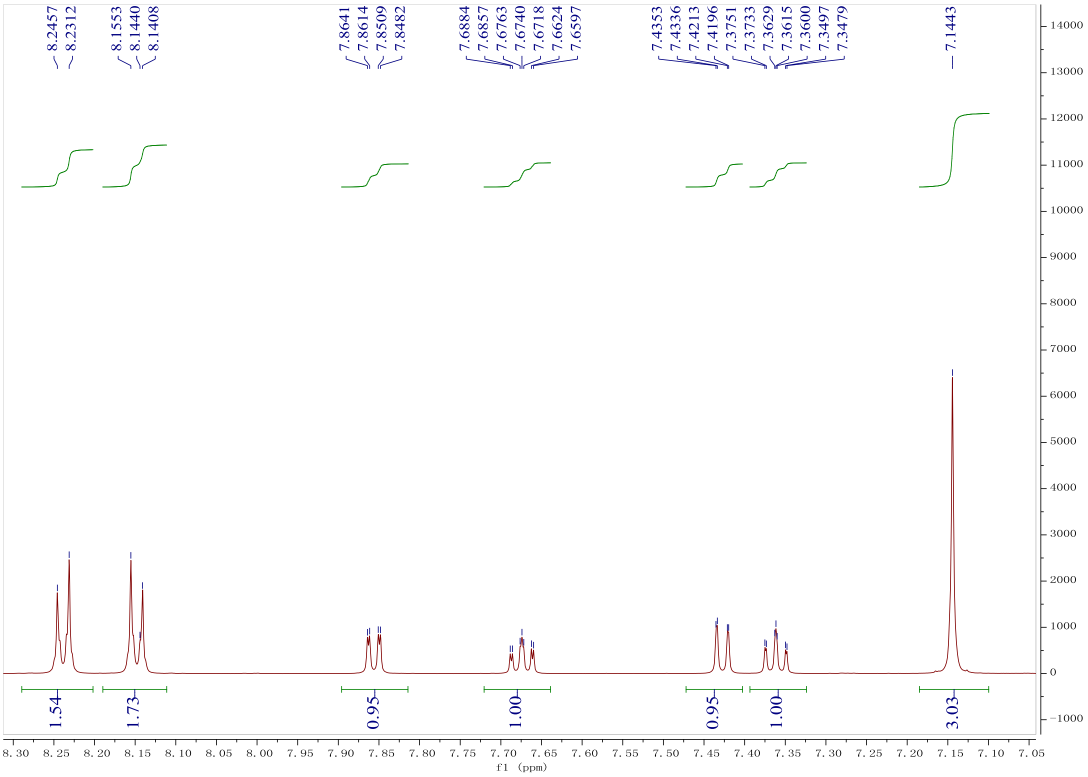
8.2457
8.2312
8.1553
8.1440
8.1408
7.8641
7.8614
7.8509
7.8482
7.6884
7.6857
7.6763
7.6740
7.6718
7.6624
7.6597
7.4353
7.4336
7.4213
7.4196
7.3751
7.3733
7.3629
7.3615
7.3600
7.3497
7.3479
7.1443
1.54
1.73
0.95
1.00
0.95
1.00
3.03
f1 (ppm)

^{13}C NMR of 5j

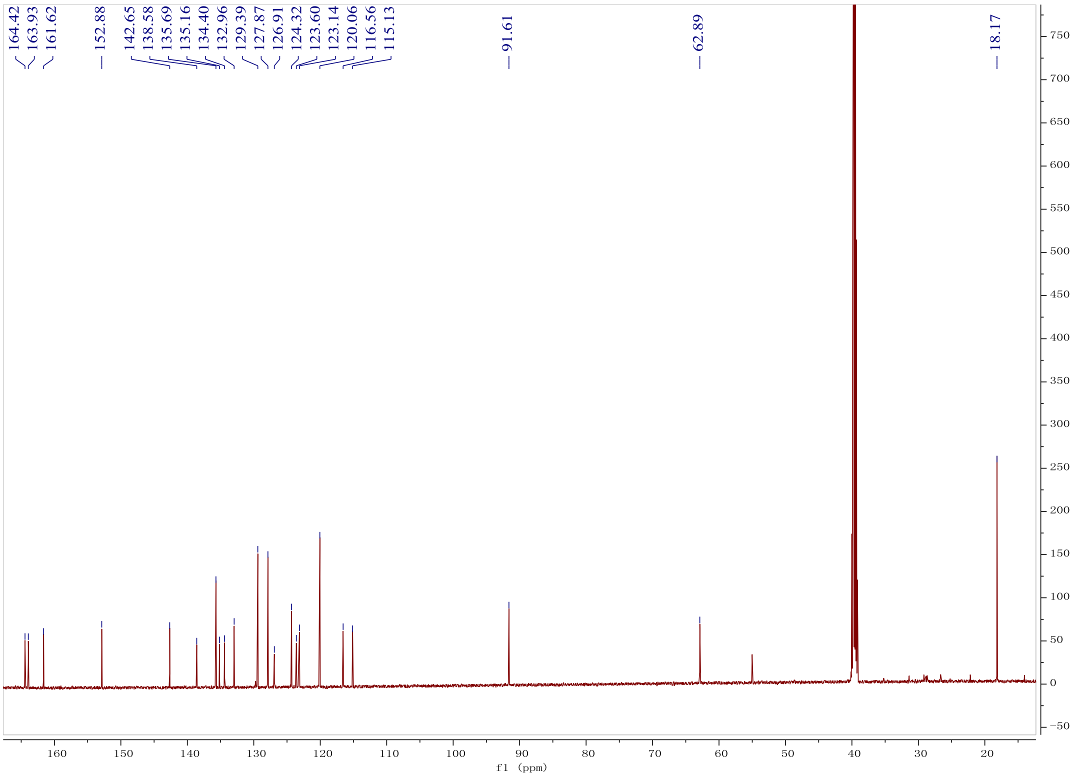
164.42
163.93
161.62
152.88
142.65
138.58
135.69
135.16
134.40
132.96
129.39
127.87
126.91
124.32
123.60
123.14
120.06
116.56
115.13
91.61
62.89
18.17
f1 (ppm)

5k

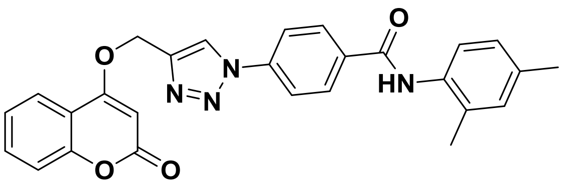

***N*-(2,4-dimethylphenyl)-4-(4-(((2-oxo-2*H*-chromen-4-yl)oxy)methyl)-1*H*-1,2,3-triazol-1-yl)benzamide**

HRMS of 5k

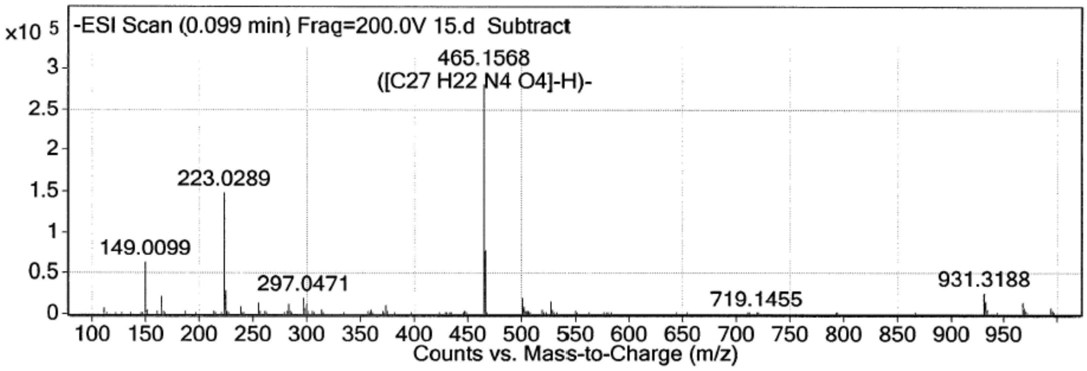

^{1}H NMR of 5k

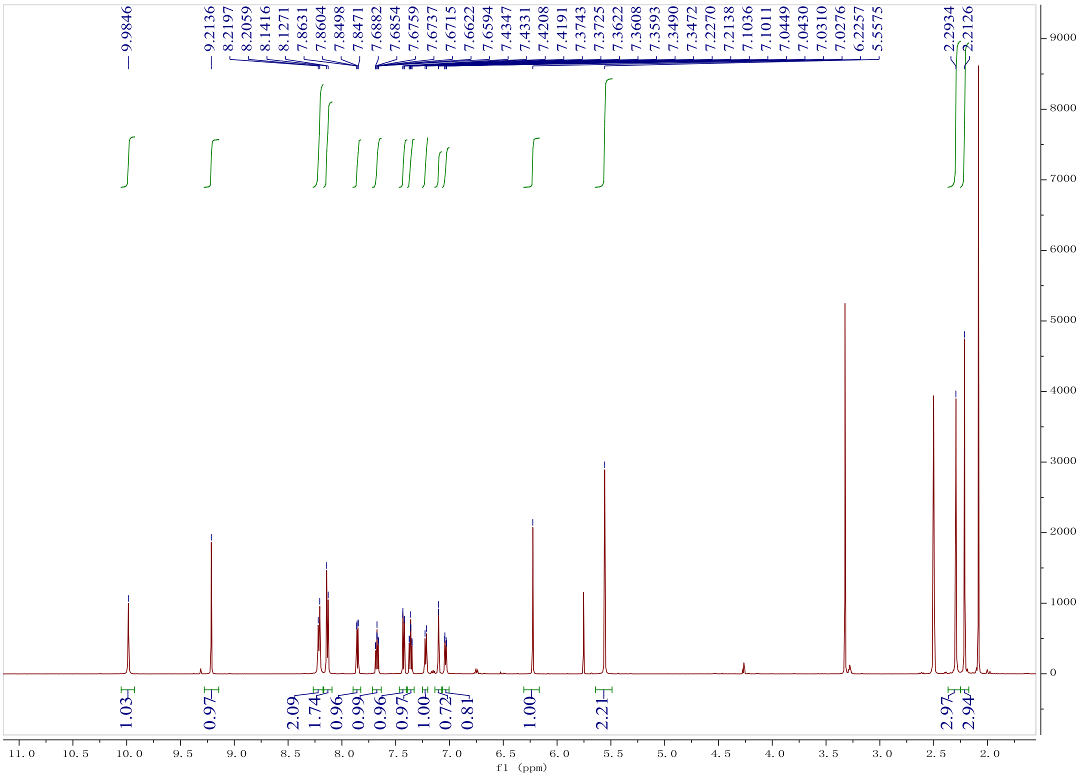

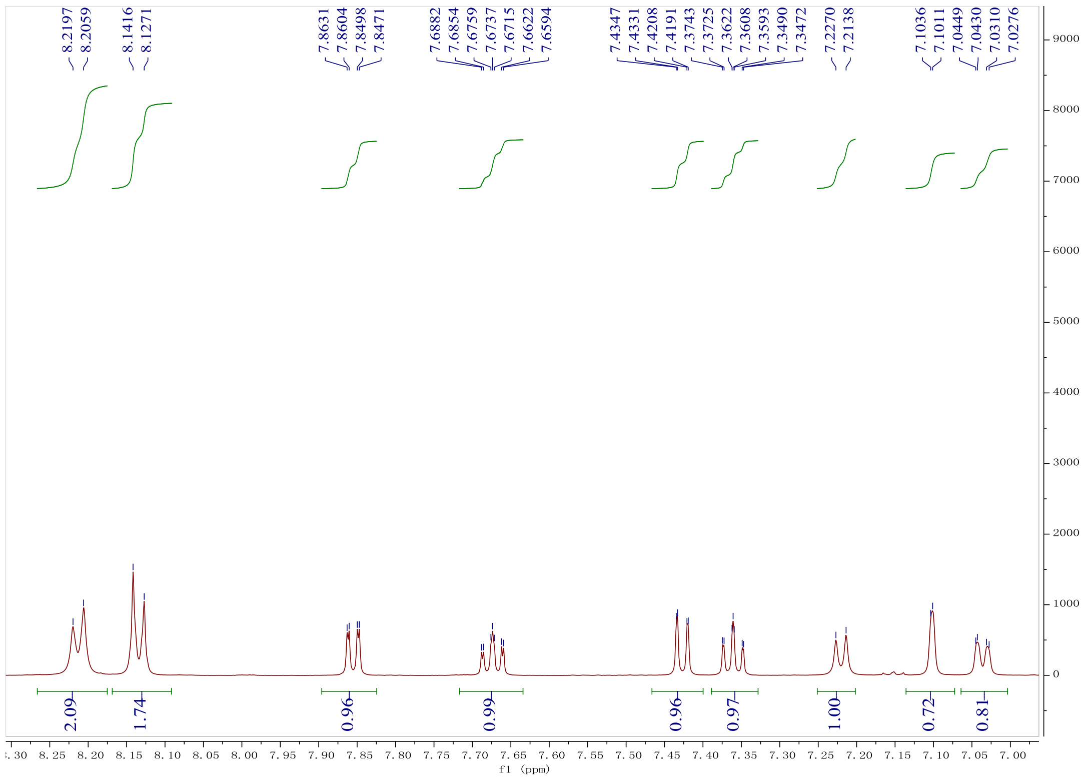

^{13}C NMR of 5k

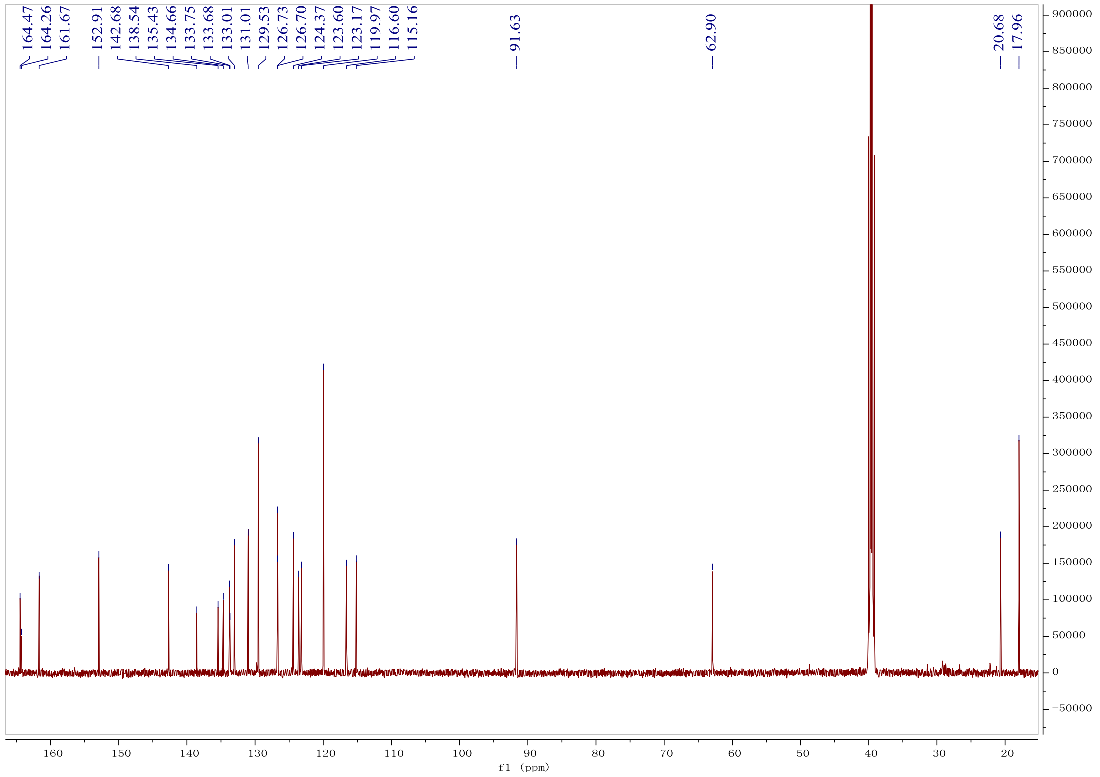

5l

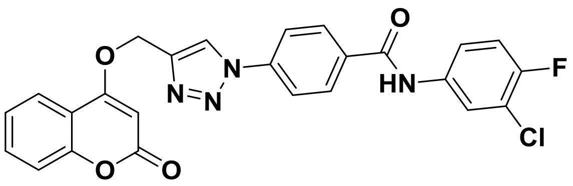

***N*-(3-chloro-4-fluorophenyl)-4-(4-(((2-oxo-2*H*-chromen-4-yl)oxy)methyl)-1*H*-1,2,3-triazol-1-yl)benzamide**

HRMS of 5l

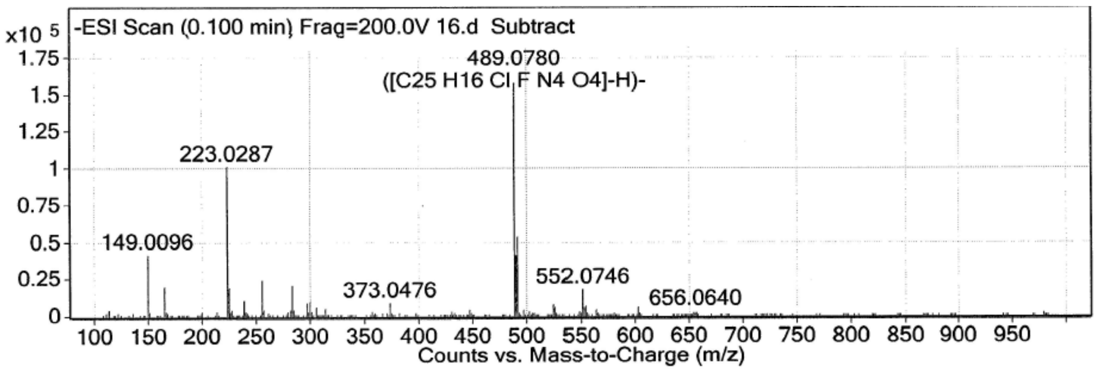

^{1}H NMR of 5l

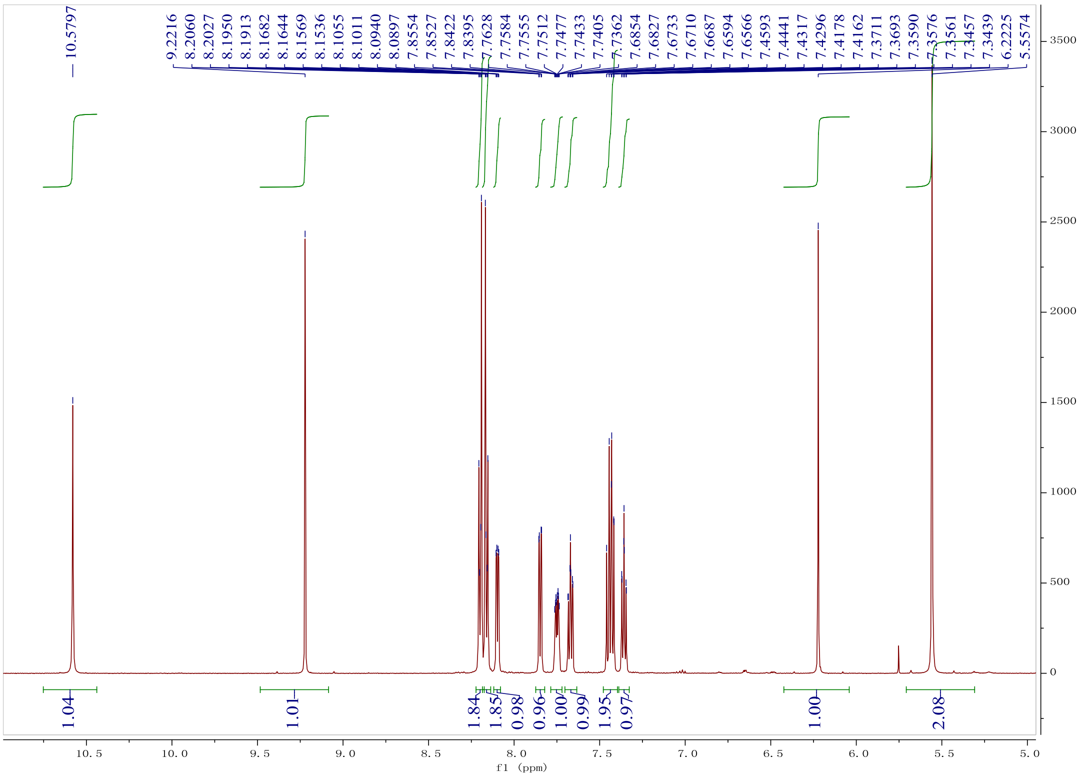

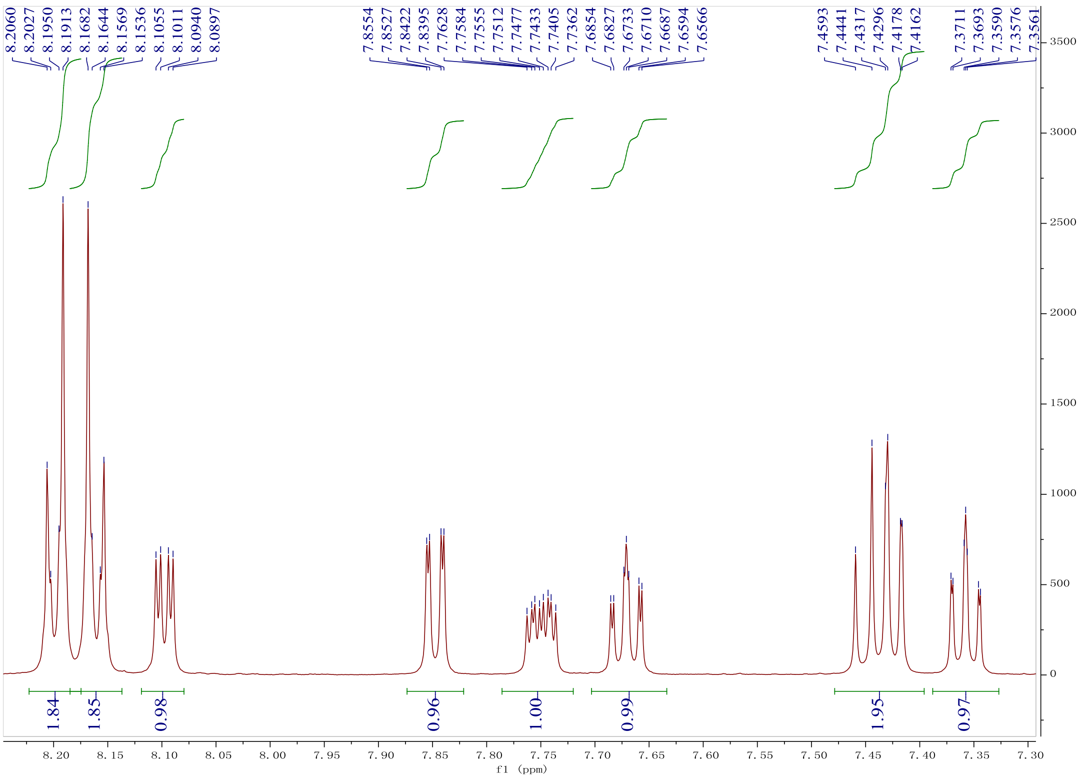

^{13}C NMR of 5l

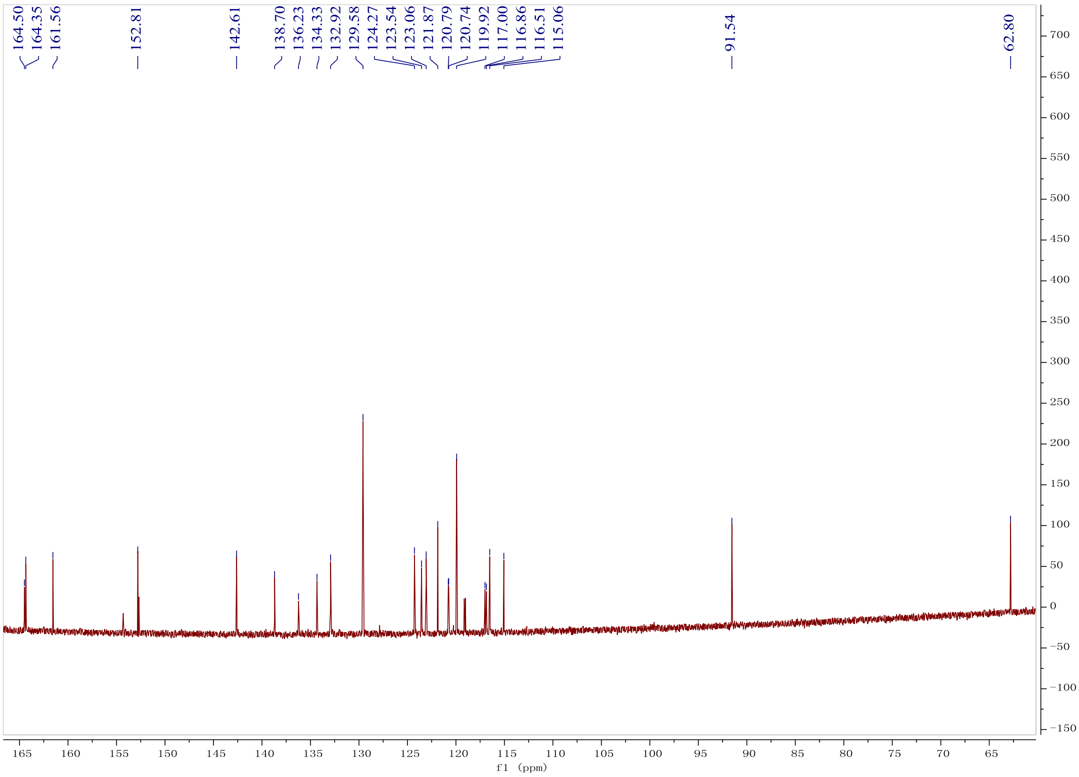

5m

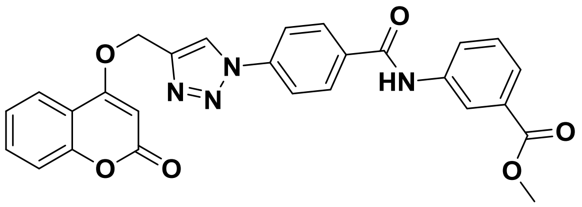

methyl 3-(4-(4-(((2-oxo-2*H*-chromen-4-yl)oxy)methyl)-1*H*-1,2,3-triazol-1-yl)benzamido)benzoate

HRMS of 5m

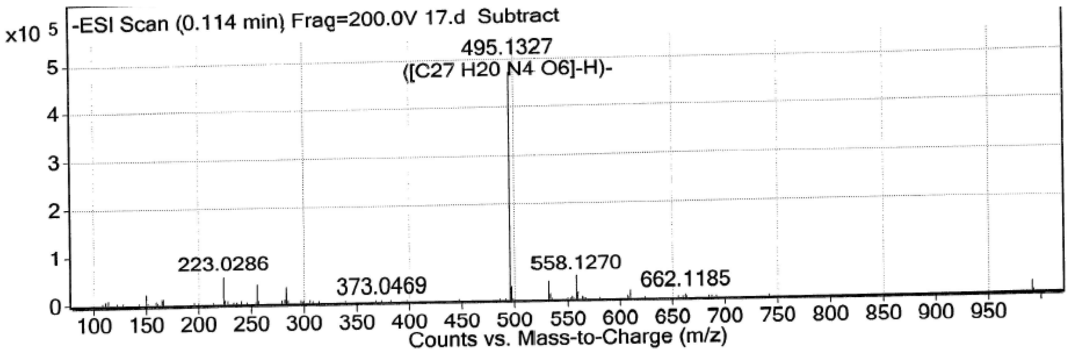

^{1}H NMR of 5m

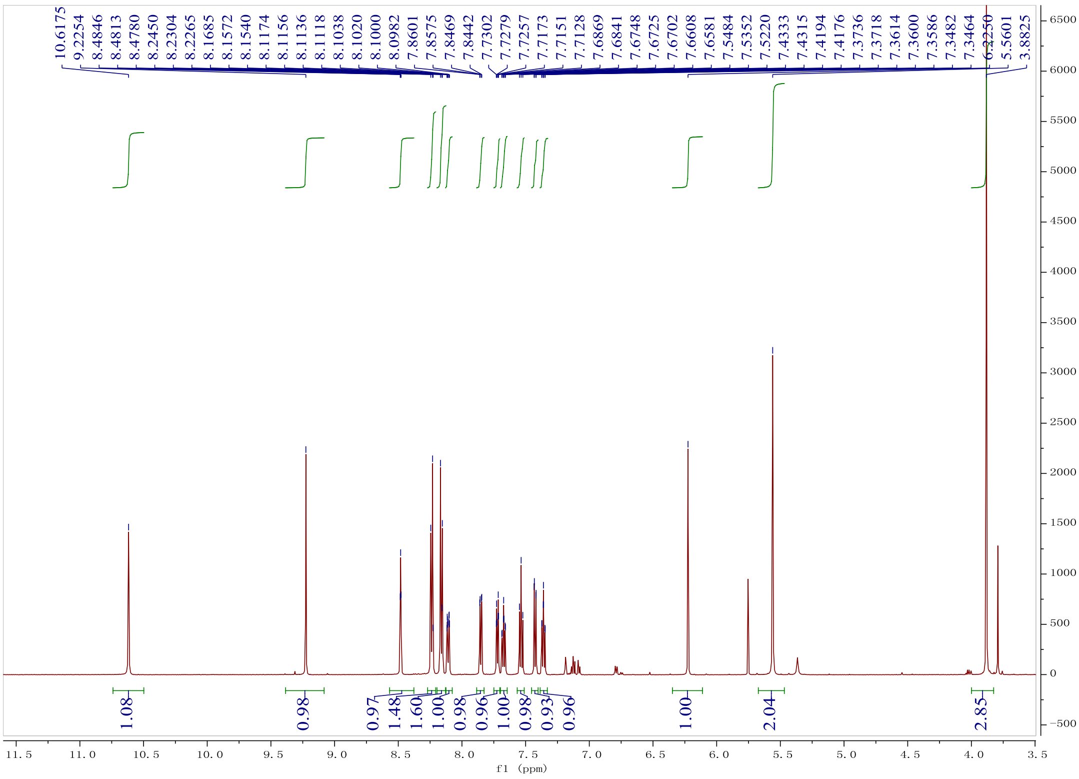

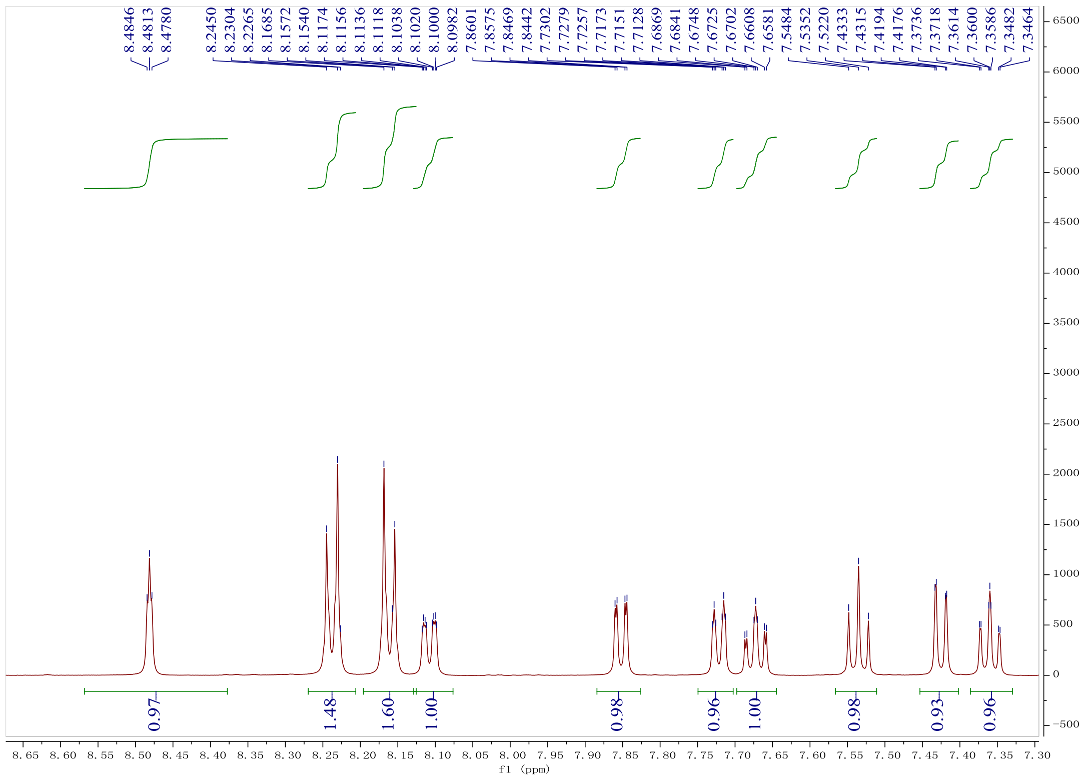

^{13}C NMR of 5m

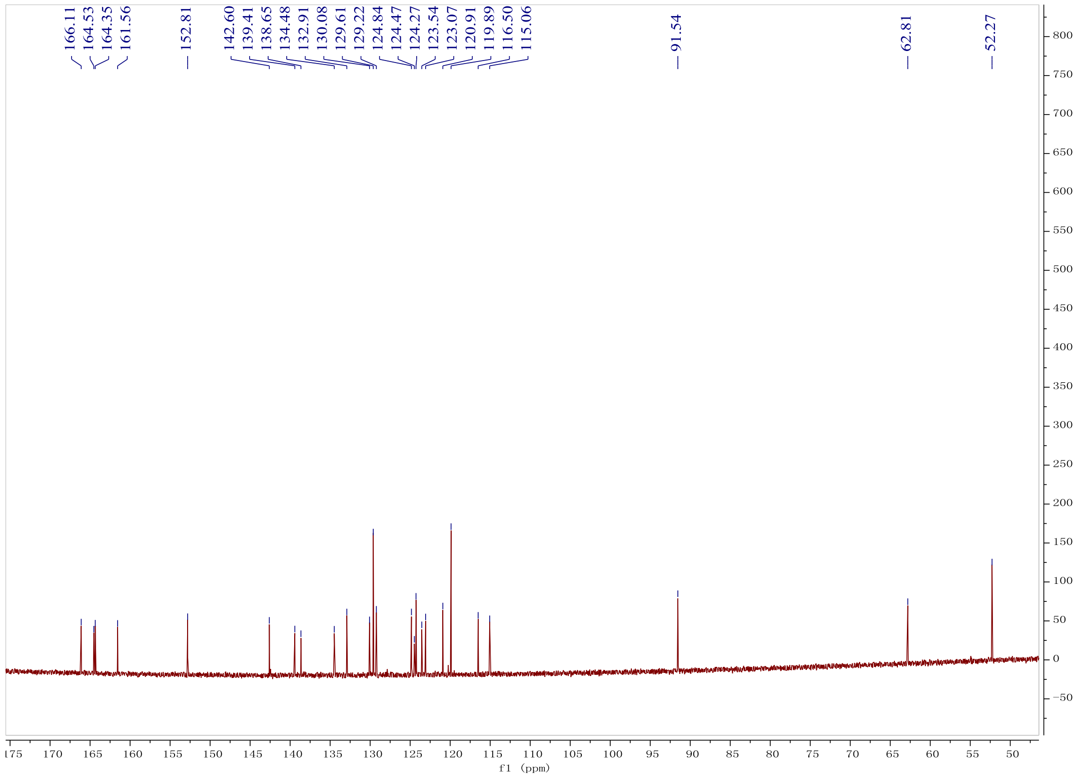

5n

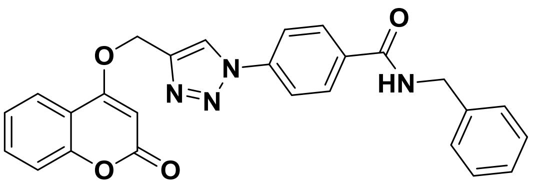

***N*-benzyl-4-(4-(((2-oxo-2*H*-chromen-4-yl)oxy)methyl)-1*H*-1,2,3-triazol-1-yl)benzamide**

HRMS of 5n

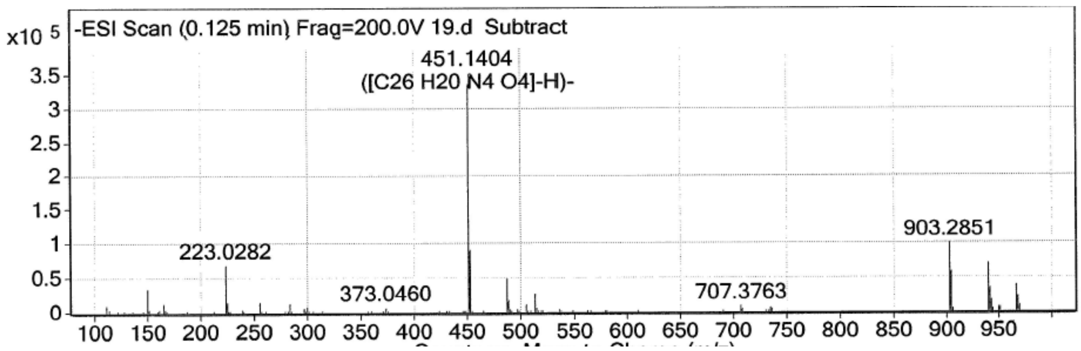

^{1}H NMR of 5n

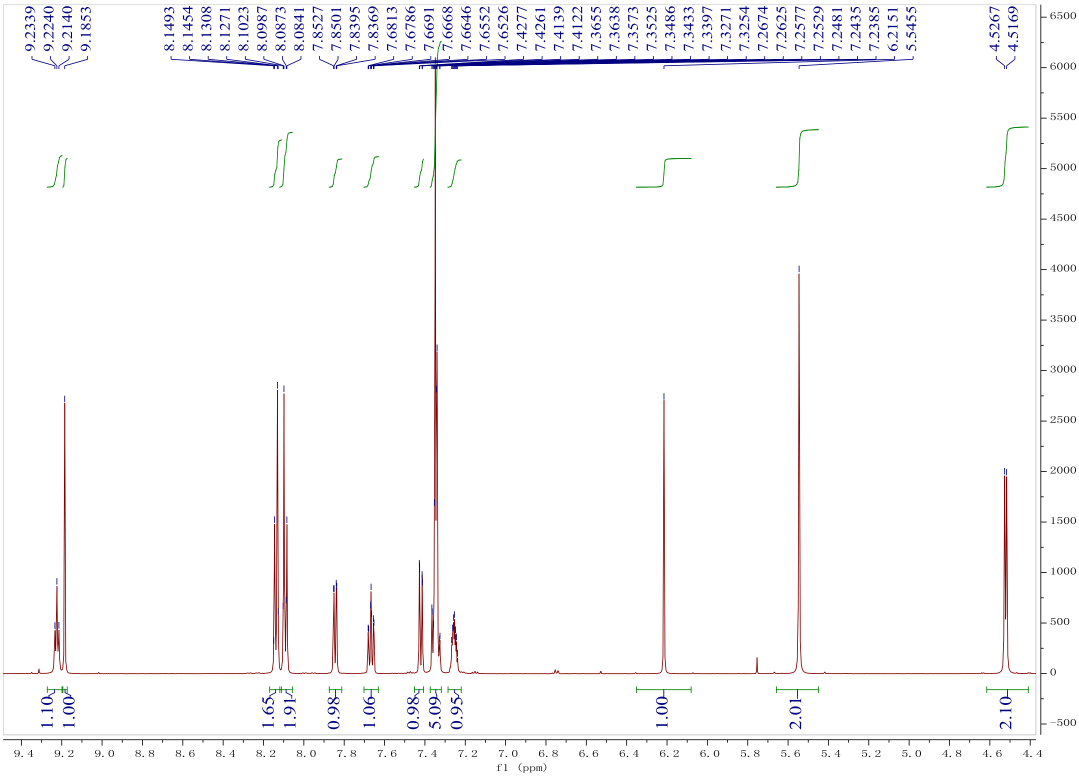

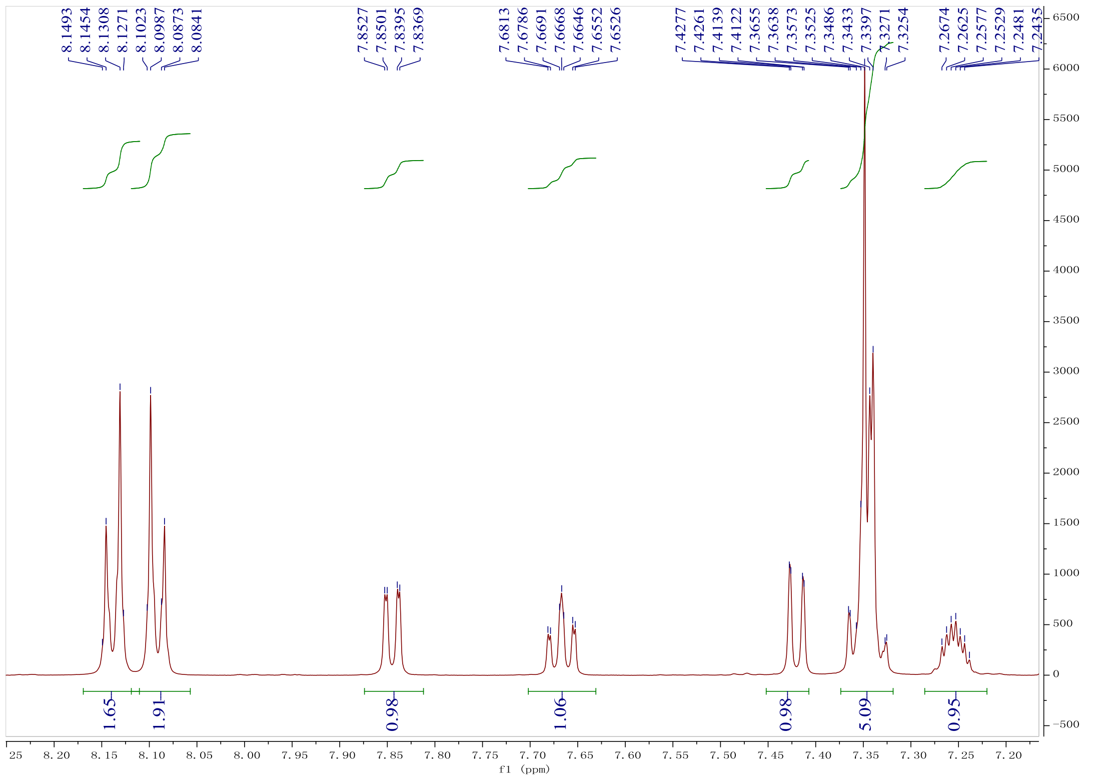
8.1493
8.1454
8.1308
8.1271
8.1023
8.0987
8.0873
8.0841
7.8527
7.8501
7.8395
7.8369
7.6813
7.6786
7.6691
7.6668
7.6646
7.6552
7.6526
7.4277
7.4261
7.4139
7.4122
7.3655
7.3638
7.3573
7.3525
7.3486
7.3433
7.3397
7.3271
7.3254
7.2674
7.2625
7.2577
7.2529
7.2481
7.2435
1.65
1.91
0.98
1.06
0.98
5.09
0.95
f1 (ppm)

^{13}C NMR of 5n

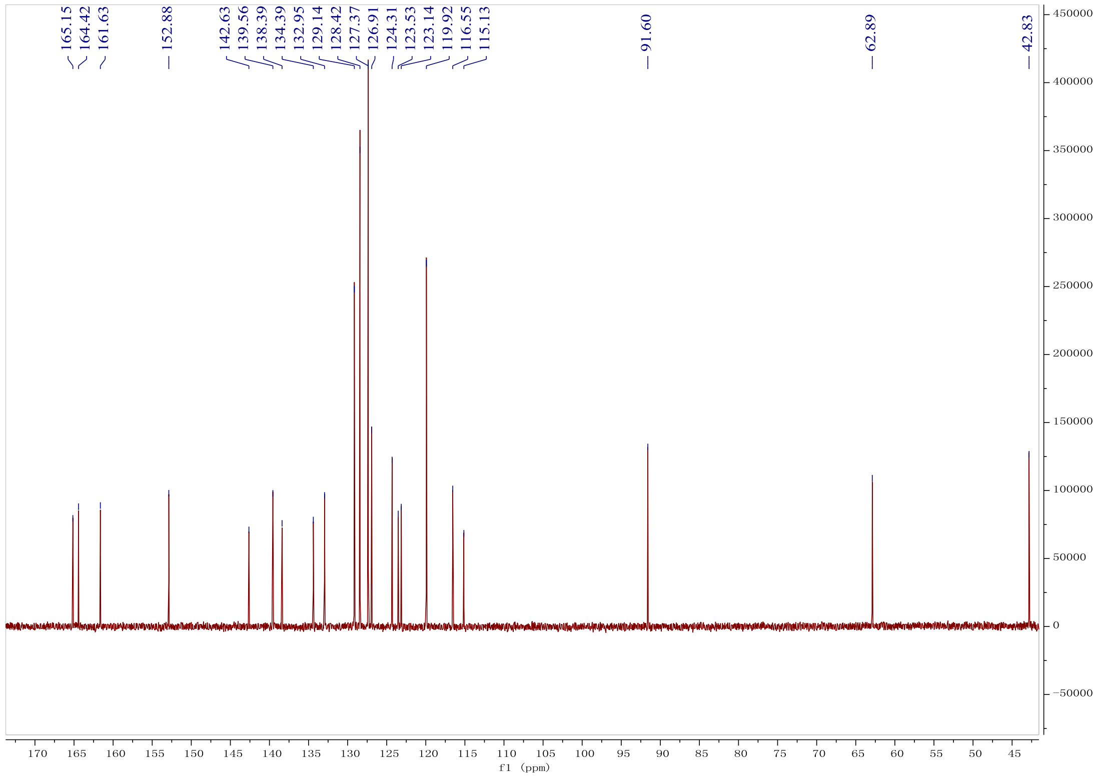
165.15
164.42
161.63
152.88
142.63
139.56
138.39
134.39
132.95
129.14
128.42
127.37
126.91
124.31
123.53
123.14
119.92
116.55
115.13
91.60
62.89
42.83
f1 (ppm)

5o

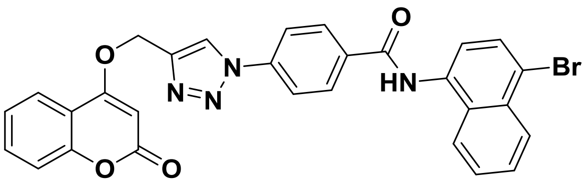

***N*-(4-bromonaphthalen-1-yl)-4-(4-(((2-oxo-2*H*-chromen-4-yl)oxy)methyl)-1*H*-1,2,3-triazol-1-yl)benzamide**

HRMS of 5o

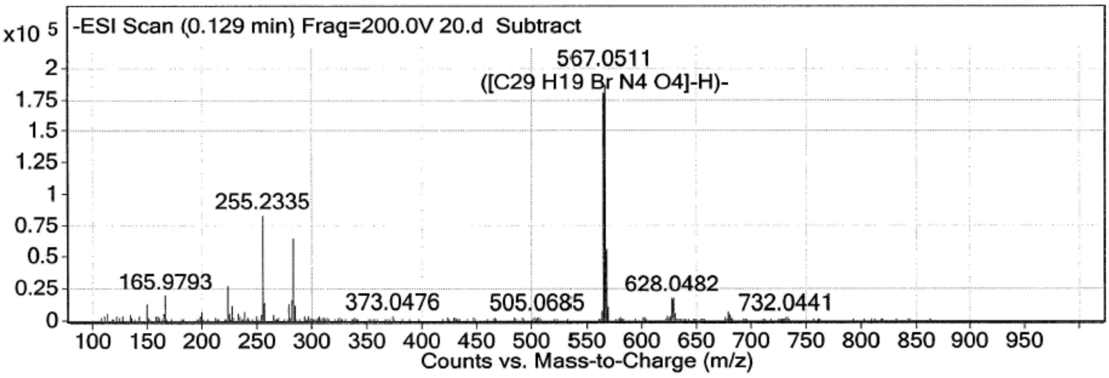

^{1}H NMR of 5o

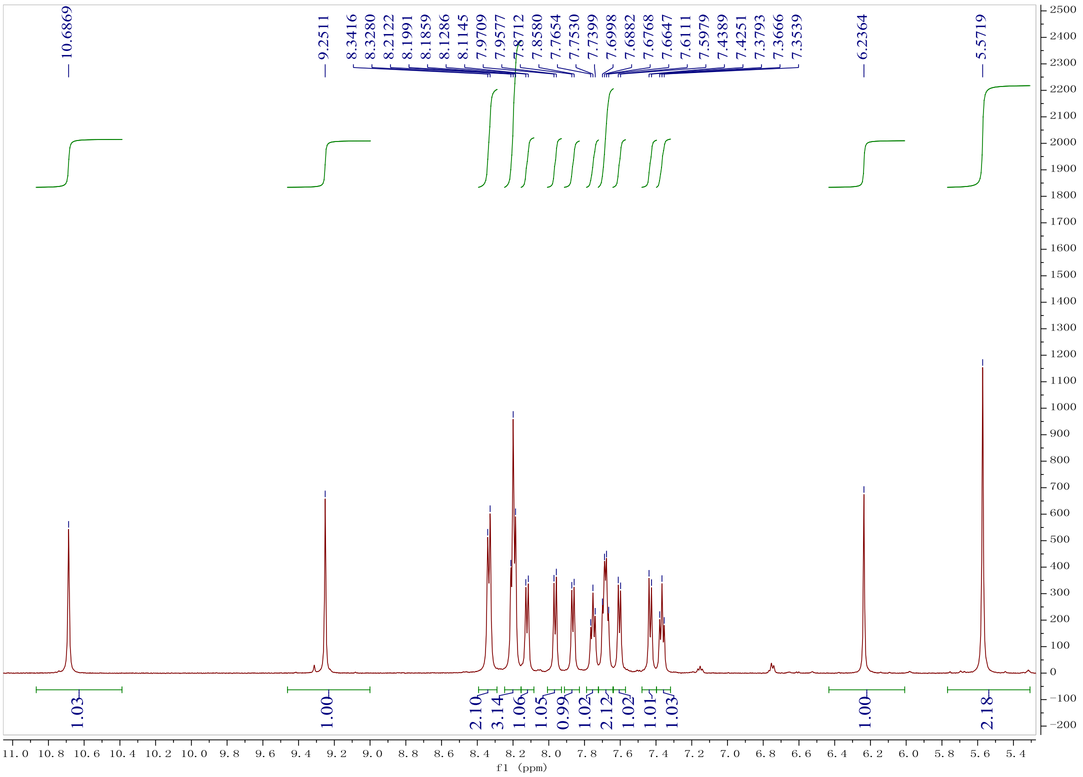

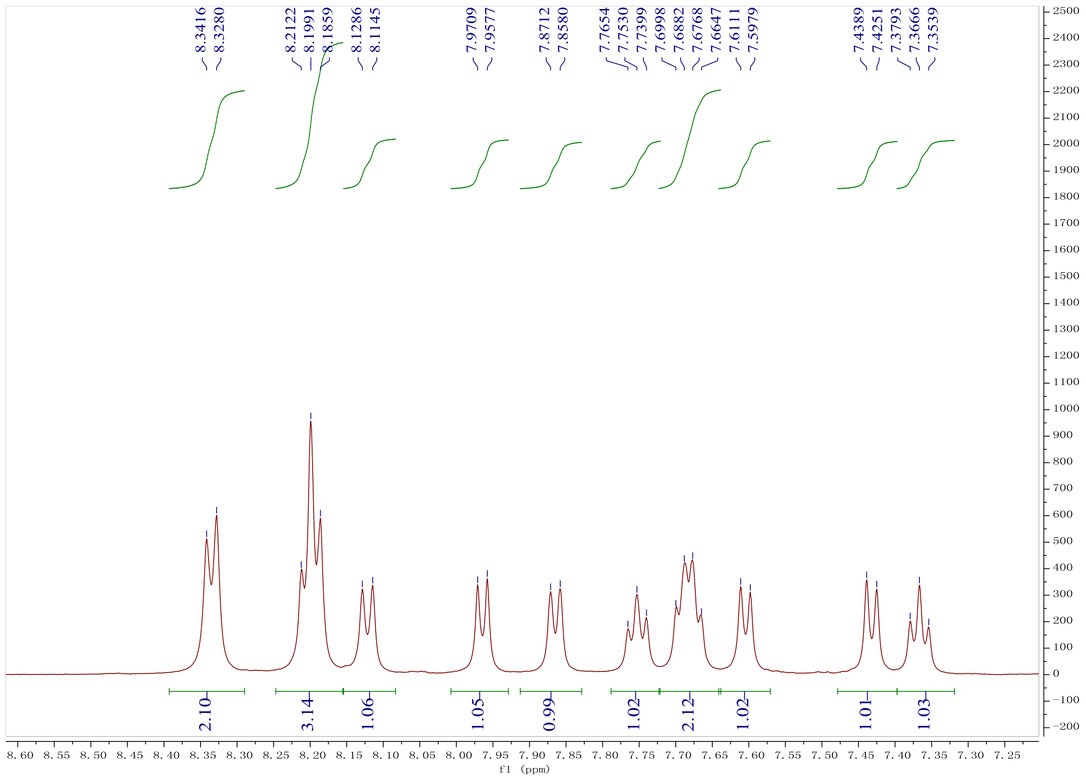
8.3416
8.3280
8.2122
8.1991
8.1859
8.1286
8.1145
7.9709
7.9577
7.8712
7.8580
7.7654
7.7530
7.7399
7.6998
7.6882
7.6768
7.6647
7.6111
7.5979
7.4389
7.4251
7.3793
7.3666
7.3539
2.10
3.14
1.06
1.05
0.99
1.02
2.12
1.02
1.01
1.03
f1 (ppm)

^{13}C NMR of 5o

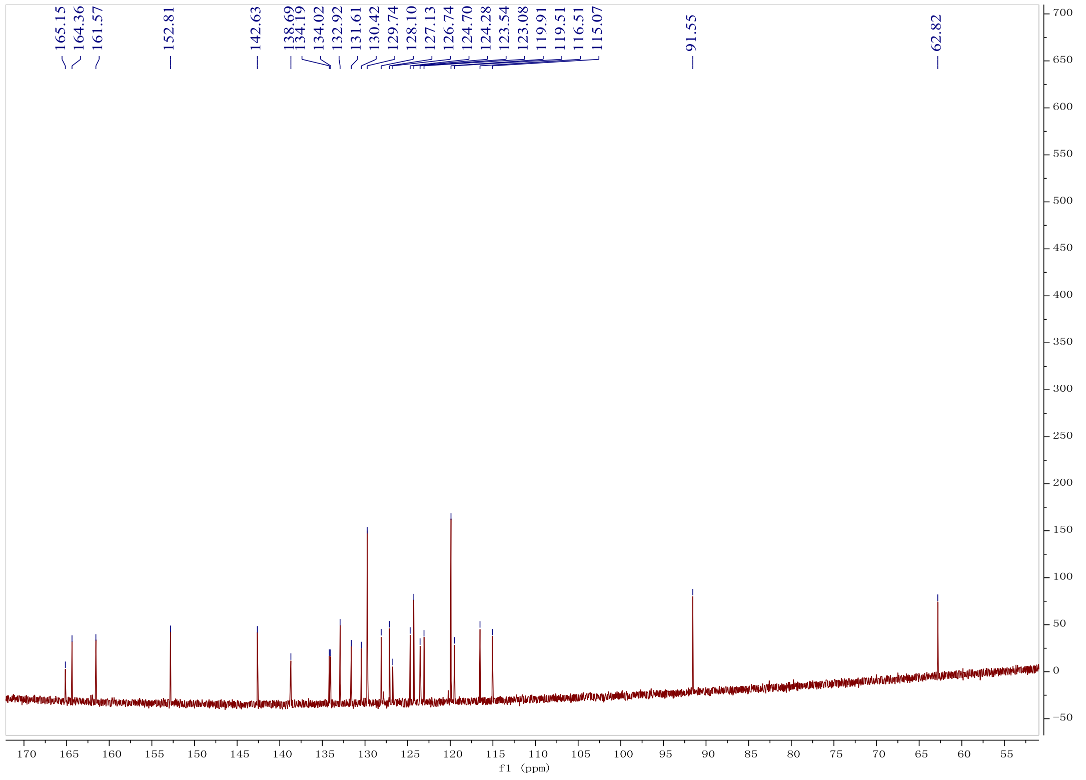
165.15
164.36
161.57
152.81
142.63
138.69
134.19
134.02
132.92
131.61
130.42
129.74
128.10
127.13
126.74
124.70
124.28
123.54
123.08
119.91
119.51
116.51
115.07
91.55
62.82
f1 (ppm)
